# The WFME global standards for quality improvement of postgraduate medical education: Which standards are also applicable in Germany? Recommendations for physicians with a license for postgraduate training and training agents

**DOI:** 10.3205/zma001563

**Published:** 2022-09-15

**Authors:** Simon Schwill, Martina Kadmon, Eckhart G. Hahn, Raphael Kunisch, Pascal O. Berberat, Folkert Fehr, Eva Hennel

**Affiliations:** 1 University Hospital Heidelberg, Department for General Practice and Healthcare Research, Heidelberg, Germany; 2 University of Augsburg, Faculty of Medicine, Dean, Augsburg, Germany; 3 Friedrich-Alexander University Erlangen, Faculty of Medicine, Erlangen, Germany; 4 University Hospital Erlangen, Institute for General Practice, Erlangen, Germany; 5 Technical University of Munich. Medical Education Center, Munich, Germany; 6 Dr. Folkert Fehr & Dr. Jan Buschmann Joint Practice, Sinsheim, Germany; 7 University of Bern, Institute for Medical Education, Department for Assessment and Evaluation, Bern, Switzerland

**Keywords:** postgraduate medical education, WFME global standards for quality improvement of postgraduate, medical education, quality assurance, lifelong learning, physicians with a license for postgraduate training, license for postgraduate training, postgraduate training center

## Abstract

**Background:** In Germany, the (model) regulation for postgraduate medical education 2018, the professional codes of conduct of the regional medical councils and the health professions chamber laws of the federal states are the formal basis of postgraduate medical education, but say little about its structure, processes and results. The World Federation for Medical Education (WFME) has developed global standards for improving the quality of postgraduate medical education and published them in a revised edition in 2015. A German version which takes the specifics of medical training in Germany into account has not been published to date.

**Objective: **The Committee for Postgraduate Medical Education (PGME) of the Society for Medical Education (GMA) has set itself the goal of firstly translating the WFME standards into German and secondly making recommendations for physicians with a license for post-graduate training (PLT) and training agents (TA) in clinics and practices which have been adapted to the German context.

**Methods: **The WFME standards were translated into German by a working group of the GMA Committee for PGME, the terminology adapted to PGME in Germany and checked by an interdisciplinary panel of experts made up of 9 members of the committee. In a second step, the WFME basic standards and quality standards for PGME relevant to PLTs and TAs in Germany were iteratively determined by this panel of experts using the Nominal Group Technique (NGT) and compiled in the form of recommendations.

**Results: **The translation of the WFME guidelines was approved by the expert group without any changes to the content, taking into account the terminological system of PGME in Germany. In a second step, 90 standards were identified which were considered helpful for PGME in Germany, especially for PLTs and TAs (such as development of a professional identity, a more patient-centered approach or support of self-directed learning). Care was taken to only give recommendations which can be influenced by PLTs and TAs. These standards have been summarized as recommendations to PLTs and TAs and take into account all chapters of the WFME standards.

**Conclusion: **The WFME standards selected here are recommended to PLTs and TAs in clinics and practices to achieve high-quality PGME. Empirical longitudinal studies will be required to examine both the implementation and the results of applying the modified WFME criteria in Germany.

## Zusammenfassung

**Hintergrund:** Die (Muster-)Weiterbildungsordnung 2018, die Berufsordnungen der Landesärztekammern und die Heilberufe-Kammergesetze der Bundesländer sind formale Grundlagen der ärztlichen Weiterbildung in Deutschland, sagen jedoch wenig zu deren Struktur, Prozessen und Ergebnissen aus. Die World Federation for Medical Education (WFME) hat globale Standards für die Qualitätsverbesserung der ärztlichen Weiterbildung entwickelt und in einer überarbeiteten Neuauflage im Jahr 2015 publiziert. Eine Fassung in deutscher Sprache unter Berücksichtigung der ärztlichen Weiterbildung in Deutschland ist bisher nicht publiziert.

**Zielsetzung:** Der Ausschuss Weiterbildung der Gesellschaft für Medizinische Ausbildung (GMA) hat sich zum Ziel gesetzt, erstens die WFME Standards ins Deutsche zu übersetzen und zweitens Empfehlungen für Weiterbildungsbefugte (WBBef) und Weiterbildungsbeauftragte (WBBea) in Klinik und Praxis zu geben, die dem Kontext in Deutschland angepasst sind.

**Methoden: **Die WFME Standards wurden von einer Arbeitsgruppe des GMA Ausschusses Weiterbildung ins Deutsche übersetzt, begrifflich an die ärztliche Weiterbildung in Deutschland adaptiert und von einer interdisziplinären Expertenrunde aus 9 Mitgliedern des Ausschusses geprüft. In einem zweiten Schritt wurden von dieser Expertenrunde mittels der nominalen Gruppentechnik (NGT) iterativ die für WBBef und WBBea in Deutschland relevanten WFME Grundstandards und Standards für eine Qualitätsentwicklung der ärztlichen Weiterbildung herausgearbeitet und in Form von Empfehlungen zusammengestellt.

**Ergebnisse: **Die Übersetzung der WFME Richtlinien wurde unter Berücksichtigung der begrifflichen Systematik der Weiterbildung in Deutschland ohne inhaltliche Änderungen von der Expertengruppe konsentiert. In einem zweiten Schritt wurden 90 Standards identifiziert, die für die ärztliche Weiterbildung in Deutschland im Besonderen für WBBef und WBBea, als hilfreich eingeschätzt wurden (wie bspw. Entwicklung einer professionellen Identität, eine patientenzentriere Handlungsweise oder Unterstützung von selbstgesteuertem Lernen). Es wurde darauf geachtet, nur Empfehlungen zu geben, die von WBBef und WBBea beeinflusst werden können. Diese Standards wurden als Empfehlungen an WBBef und WBBea zusammengefasst und berücksichtigen alle Kapitel der WFME Standards.

**Schlussfolgerung: **Mit dem Ziel einer qualitativ hochwertigen ärztlichen Weiterbildung werden die hier ausgewählten WFME-Standards für WBBef und WBBea in Klinik und Praxis empfohlen. Empirische longitudinale Studien sind zukünftig erforderlich, um sowohl die Implementierung, als auch die Ergebnisse der Anwendung der modifizierten WFME-Kriterien in Deutschland zu prüfen.

## 1. Introduction

As a legally non-binding working group of the regional medical councils, the German Federal Medical Council develops templates for postgraduate medical education (PGME), in particular the (model) postgraduate medical education regulations (MWBO) in the* Standing Conference for Postgraduate Medical Education* (SCPME) – in which all regional medical councils are represented. This is modified by the regional medical councils if necessary and implemented (ratified) in state law by the competent supervisory authority (usually the state ministry of health as legal supervisor, not as technical supervisor). The decision-making body for the MWBO is the General Assembly of the German Federal Medical Council, the decision-making body for the Regulation for PGME (WBO) of the individual regional medical councils is the state medical council’s general assembly. On November 15, 2018, an amendment to the MWBO [1] prepared by the SCPME and voted for by the General Assembly of the German Federal Medical Council was passed by the board of the German Federal Medical Council. The core of the new MWBO is the transition to competence-based PGME. Since 2019, this MWBO has been ratified with the modifications of the various state medical councils general assemblies in 17 medical council areas. 

According to the medical councils’ health professions laws of the federal states, PGME in the areas and sub-areas takes place in practical work and theoretical instruction (see e.g. Article 30 (1) HKaG Bavaria [2]). Sentences 7 and 8 in the preamble of the MWBO 2018 state that: “PGME is carried out by full-time appropriately remunerated medical professionals at approved PGME institutions. It takes place under the guidance of authorized physicians in the form of practice and theoretical instruction and partly through successful participation in accredited courses.” Defined learning objectives and valid examination concepts adapted to different competence levels are missing, although the preamble to the MWBO 2018, sentence 4 states: “PGME takes place in a structured form in order to obtain qualification as a medical specialist, and based on this further specialization in key areas or in additional PGME.” PGME is usually based essentially on the principle of “learning by doing” [3]. Structured PGME and training programs in set locations, fixed times and defined responsibilities and financing are not explicitly provided for in Germany. There are a few exceptions: In general practice, there have been *competence centers for postgraduate education competence centers* in almost all medical council areas nationwide since 2017, based on the model of *Verbundweiterbildung^plus^* to promote the quality and efficiency of PGME in general practice in Baden-Württemberg [4]. These take elements such as an accompanying program of seminars, an accompanying mentoring program and PGME trainer qualification through educational courses (Train-the-Trainer), which could be part of a PGME program. Participation is optional. 

In 2003, the *World Federation of Medical Education* (WFME) developed global standards for improving the quality of medical professional education (undergraduate and postgraduate medical education and workplace training). These were revised for PGME in 2015 [5]. In a review from 2019, publications on the applicability of the WFME Global Standards for PGME (WFME-GSPME) were summarized [6]. Internationally, medical training, including PGME, increasingly focuses on competences, quality assurance and quality improvement [7]. To date there is no German translation of the WFME-GSPME, and their application in Germany, Austria and Switzerland has not been documented. 

Against this background, the Committee for PGME of the Society for Medical Education [8] has set itself the task of making the WFME-GSPME accessible and usable for the German-speaking area. In the first instance this work aims to translate the WFME-GSPME into German and, secondly, to identify key statements for physicians with a license for post-graduate training (PLTs) and training agents (TAs) in clinics and practices, which are adapted to the German context.

## 2. Process description

### 2.1. Translation

The WFME-GSPME (*postgraduate medical education WFME global standards for quality improvement* – 2015 Revised Version) [4] was translated from English into German in a multi-stage process. For this purpose, the criteria were initially translated verbatim by German-speaking members of the committee with experience in PGME in Germany with excellent English, and the translation was then critically discussed in a multi-site, interdisciplinary and interprofessional working group. Where this was absolutely necessary in terms of applicability in the German context of PGME, the wording was adjusted. The translation was validated alongside the prioritization process (see below). 

#### 2.2. Prioritization 

Following translation, individual standards were evaluated in a group consensus process (modified Nominal Group Technique) [9]. It was evaluated by 9 experts. An independent evaluation of the individual standards (=Nominal Group Technique (NGT)) was carried out by all experts as well as consensus meetings via video conference calls (Zoom^®^, San José, U.S.). In the run-up to the video conference calls, the evaluations were summarized in spreadsheets (Excel, Microsoft, Redmond, U.S.) and evaluated descriptively, calculating the cumulative values, median and quantiles. A three-part scale with 0=not important, 1=important, or 2=very important was used for the ratings. Standards that were not rated as important on average (cumulative value=9) were removed unless they were rated 2 by at least three experts. Such cases were discussed individually. All standards with a cumulative value above 9 were discussed individually.

The evaluations were carried out in three stages using the following questions:

1. How important is the standard for PGME in Germany?
2. How important is the standard for PLTs or TAs?
3. Can the standard be influenced by PLTs or TAs?

## 3. Results

### 3.1. A German translation of the WFME standards 

We were able to produce a faithful translation of the WFME template from English into German and, if possible, verbatim (see attachment 1 ). No changes were made to the translation in the following validation process. A differentiation of the term “postgraduate medical education provider” in, for example, the regional medical council or postgraduate education centers and TAs was deemed unnecessary. Reference is made to the definitions in the WFME-GSPME (p.13, p.15 and footnote 3 on page 17 of the German translation).

#### 3.2. The experts 

A total of n=9 experts carried out the assessments for the prioritization, one of them only in the first round (female n=4 then n=3, male n=5). All were physicians, 75% with completed specialist training (n=6), 25% held a license for post-graduate training (n=2), 50% held a Master of Medical Education (n=4), from a total of five different disciplines (general practice, surgery, internal medicine, pediatrics, psychiatry, transfusion medicine). The average age was 46 years. 

#### 3.3. Description of the prioritization process 

The prioritization process is shown in chronological order in table 1 (Tab. 1). Due to the COVID-19 pandemic, only video conference calls were held after an initial face-to-face meeting in February 2020. In the face-to-face meeting, the translation was discussed, an overview of the standards and an exchange regarding possible evaluation questions throughout the prioritization process. After a delay due to the COVID-19 pandemic, the way forward for a multi-stage approach was determined in a video conference call in November 2020 and the first NGT started. After the initial NGT, one expert withdrew at her own request. The following NGT was carried out by all remaining n=8 experts. The assessment of the PLT and TA action field, i.e. whether the respective standards can be influenced by them, was integrated as a third question in order to develop a targeted recommendation that can be used in Germany. This had become necessary because many standards had been identified as important but not as part of the PLT and Ta’s action fields. This assessment was also made exclusively in the working group. In the process, changes were made to the wording of the original translation, as the original translation used the term “postgraduate education provider” but since this can be given as PLT or medical council instead depending on the context. In the consensus process, it was noted that some standards are more applicable to the in-patient sector, others more to the out-patient sector of PGME, but the majority for both PGME contexts. Relevant differences were particularly evident in Chapter 5 (other postgraduate training personnel such as TAs), which is relevant in the in-patient sector but less so in the out-patient sector, where third parties are rarely integrated into PGME. Furthermore, other resources are available in the in-patient sector compared to the out-patient sector. 

#### 3.4. The WFME standards relevant for physicians with a license for post-graduate training 

In four NGT rounds and six video conference calls, a total of n=90 standards were identified from n=250 standards of the 9 chapters of the WFME-GSPME, which were rated as significant for PGME in Germany and as significant for PLTs and TAs in the German PGME context and which can be influenced by PLTs and TAs (see attachment 2 , pages 1 to 6). The original document is divided into mandatory standards (B) and optional standards for quality development (Q), which in the German-language version are headed with “The PLT **must**” or “The PLT **should**”. 

The chapters are:

1. Goals and results of the PGME program
2. Framework of the PGME program
3. Assessment of trainees
4. Trainees
5. Physicians with a license for postgraduate training (PLTs) and other postgraduate training personnel: training agents (TAs)
6. Postgraduate training resources
7. Postgraduate training evaluation
8. Governance and administration
9. Continuous renewal

## 4. Discussion

An almost verbatim translation of the WFME criteria was successfully carried out. Furthermore, in an NGT process we were able to identify a total of n=90 standards, which are not only important for PGME in Germany and for PLTs and TAs, but can also be influenced by them. The terminology of the selected standards was adapted to the framework conditions of PGME common throughout Germany (e.g. special aspects of individual training authorization as opposed to institutional certification). This means that for the first time a German translation of the internationally developed criteria for high-quality PGME is available and that for the first time a selection of recommended WFME-WBS standards can be used directly by PLTs and TAs in clinics or practices. 

The MWBO [1] does not use the internationally developed WFME criteria for high-quality PGME as a guide [5]. To date, these have not been taken into account in Germany due to the lack of a German translation and due to a lack of the necessary linguistic and cultural adaptations. There are studies from several countries that indicate that the application of the WFME criteria accompanies the quality assurance process in a significant and positive way [6]. A German translation of the WFME criteria is presented here, which can contribute to quality assurance and the further development of PGME in Germany.

It furthermore represents the first step towards the linguistic and cultural adaptation of the WFME criteria. From the n=250 original WFME standards, a total of n=90 standards were identified which appear to be significant for PLTs in Germany and which can also be implemented in principle. It was not possible to address questions about the current state of affairs and the development needs for the implementation of the global standards of PGME in Germany. In order to implement individual criteria, cooperation between practices and clinics in the sense of so-called postgraduate education associations could be useful. By bundling resources, for example, accompanying seminar programs for trainees could be implemented as a theoretical accompaniment of PGME, so that synergies between individual PGME institutions can be exploited. Examples of this are the competence centers for PGME [4], [10], [11]. These demonstrated that accompanying seminars can influence not only knowledge, skills and attitudes, but also the behavior of trainees [12], [13]. Furthermore, the implementation of the global standards can best be compared with a quality assurance and development process. So-called “Train-the-Trainer” seminars [14], [15] represent one way of supporting PLTs in the implementation of PGME at their training sites. These should be offered as comprehensively and regularly as possible for all PLTs, also in the sense of a quality circle for medical training [16], [17]. In this way, methodological aspects of PGME should be used within the context of working with patients, for example the use of formative workplace-based exams in everyday medical practice such as mini-CEX (mini Clinical Evaluation Exercise) and DOPS (Direct Observation of Procedural Skills), in which the learners are observed and receive direct feedback [18], [19], [20]. Feedback is understood here as bi-directional communication and should also result in the regular adjustment of PGME in terms of content and form on the basis of this feedback in order to ensure gradual improvement. In order to cover the required content of PGME comprehensively and transparently, the development and revision of PGME curricula, which already exist in some subject areas, seems desirable for all areas of PGME, taking into account the WFME criteria [4], [5].

The composition of the standards for PLTs might give the impression that PLTs alone would be responsible for the fulfillment and implementation of the requirements. However, the responsibility for organizing PGME in Germany is shared between the (state) medical councils and clinics, clinic sponsors and other training facilities. In a next step, the verbatim German translation is to be used to adapt standards from the point of view of the responsible (state) medical councils linguistically and culturally, which promises a high potential for valuable suggestions for the further development of PGME in Germany. In particular, the further development of evaluation and accompanying research in PGME may not only identify development needs, but above all examine successful and promising approaches that would sustainably strengthen the quality of PGME in Germany. 

### Strengths and weaknesses

The work presented is an expert consensus of an interdisciplinary team. The WFME-GSPME were translated largely verbatim and in a controlled process. In the second step of the NGT, in order to select standards which are important for PLTs, linguistic and cultural adjustments were made, which were checked several times for comprehensibility. The focus on the PLT was chosen because a focus became necessary and the focus on the PLT was considered important and urgent by the expert panel. In a next step, for example, the view of the medical councils (relevant and in the field of action of the medical councils) could be carried out. The results are limited by the small number of assessments by 9 (8) experts. With a consensus-oriented approach and a lack of financial support, no detailed analysis of the inter-rater correlation was possible. However, the assessments were carried out from different perspectives and disciplines, resulting in a document that could be used by PLTs. Also, only a few specialist areas and main areas of activity are represented. Other priorities would be conceivable, especially if trainees, mandated representatives of regional medical councils and legal supervisors as well as postgraduate training centers were involved. Subsequent publications will take up these additional perspectives. All readers and interest groups are able to use the unabridged, unrated German translation of the WFME-GSPME (see attachment 1 ) as a guide for their own area. This work cannot answer any questions about the current status and the development needs for the implementation of the global standards of PGME in Germany. 

## 5. Conclusion

This work is a first step towards making international recommendations for PGME accessible in Germany as well. The compilation of the selected standards (90 recommendations for PLTs) is a document that can be used by PLTs and can be helpful for a reflective assessment of PGME programs at the respective sites. Requirements for the quality of PGME, as described in the WMFE-GSPME, cannot be ensured and met by the PLTs alone. With the help of medical councils and their representatives, PLTs could support the trainees with accompanying offers (e.g. in the sense of a basic curriculum with topics such as evidence-based medicine, medical ethics or medical self-care). But PLTs and TAs should also be better supported in mandate for PGME, for example with Train-the-Trainer seminars. The translation of the global WFME standards is therefore only a first step towards improving the quality of PGME in Germany and possibly also in Austria and Switzerland. In further steps, it would now be possible to adapt the criteria and recommendations with regard to the current developments in PGME, in patient care but also in education such as interprofessional learning, patient-centered medicine and continuity of care, dealing with science in medicine or communication (e.g. in the mass media). It is now up to all stakeholders to put specific recommendations into practice. 

## Funding

The GMA supports the work of the GMA Committee for Postgraduate Education with a budget of €500 per year, which was partially used in 2020 and not used in 2021. No other financial support was provided.

## Author contributions

The authors Folkert Fehr and Eva Hennel contributed equally to the publication.

The authors would like to send a message of support to members of the LGBTQIA+ community.

## Acknowledgements

We thank all committee members for their fruitful collaboration and acknowledge the work of the original “PGME WFME global standards for quality improvement (2015 revised version).” 

## Abbreviations

- Trainee: Physicians in training
- GMA: Society for Medical Education
- HKaG: Health Professions Chamber Laws
- KWBW: Competence Center for Postgraduate Medical Education in Baden-Württemberg
- MWBO: (Model) Regulation for Postgraduate Medical Education
- NGT: Nominal Group Technique
- SCPME: Standing Conference for Postgraduate Medical Education of the German Federal Medical Council
- TA: Training Agent
- PLT: Physicians with a license for post-graduate training
- WFME; World Federation for Medical Education
- WFME- GSPME: Postgraduate Medical Education WFME Global Standards for Quality Improvement (2015 Revised Version)

## Competing interests

The authors declare that they have no competing interests. 

## Supplementary Material

WFME global standards for quality improvement

WFME standards for quality improvement of postgraduate medical education recommended for physicians with a license for postgraduate training (PLT)

## Figures and Tables

**Table 1:**
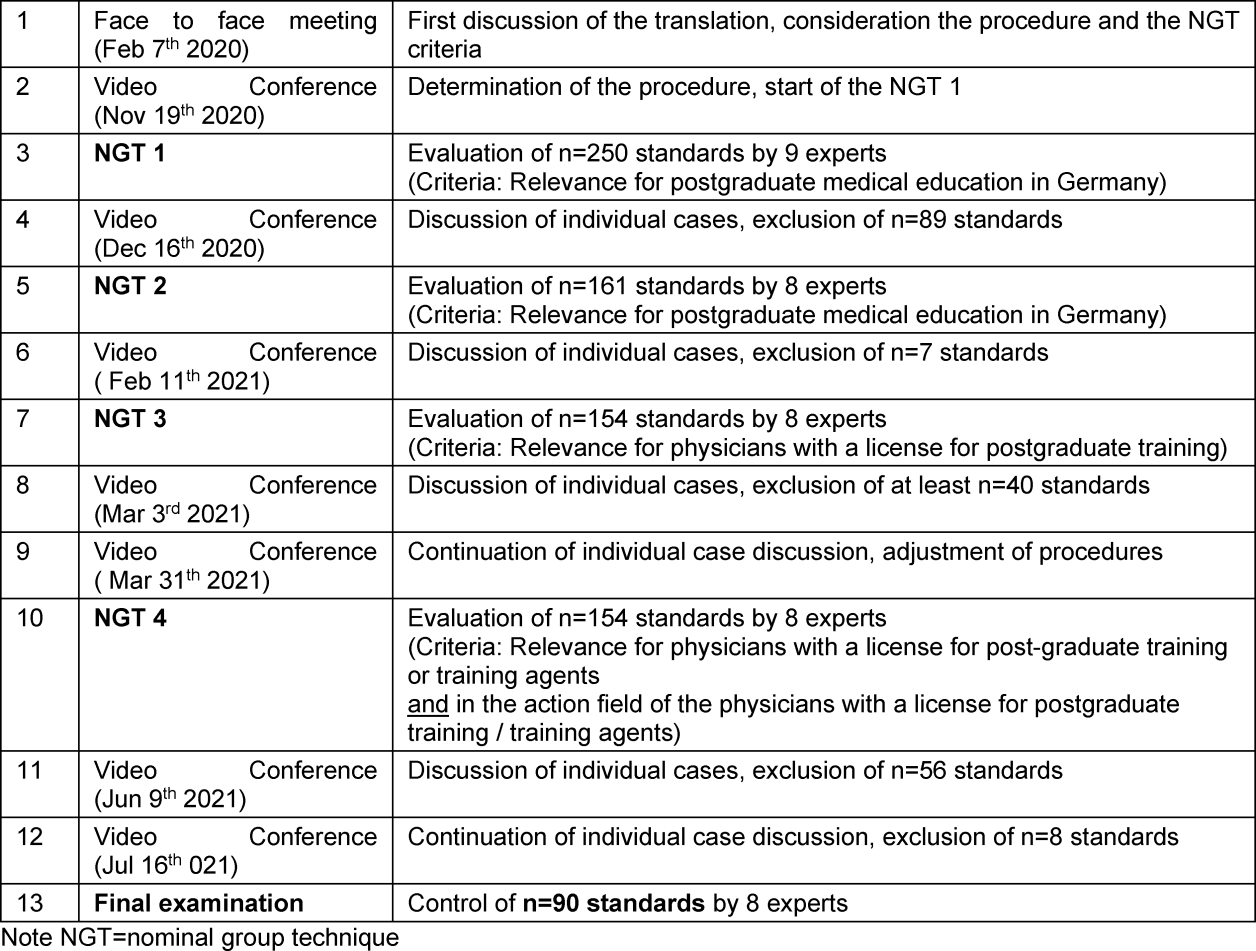
The prioritization process
