# Supplementary material for: The WFME global standards for quality improvement of postgraduate medical education: Which standards are also applicable in Germany? Recommendations for physicians with a license for postgraduate training and training agents
Source: GMS J Med Educ. 2022 Sep 15;39(4):Doc42. doi: 10.3205/zma001563 (PMC9585417; doi:10.3205/zma001563)
Supplement: WFME global standards for quality improvement [file JME-39-42-s-001.pdf]

Attachment 1: WFME global Standards for quality improvement, the 2015 revision

Attachment 1 to Schwill S, Kadmon M, Hahn EG, Kunisch R, Berberat PO, Fehr F, Hennel E. *The WFME global standards for quality improvement of postgraduate medical education: Which standards are also applicable in Germany? Recommendations for physicians with a license for postgraduate training and training agents*. GMS J Med Educ. 2022;39(4):Doc42. DOI: 10.3205/zma001563

WORLD FEDERATION FOR MEDICAL EDUCATION

POSTGRADUATE MEDICAL EDUCATION

WFME GLOBAL STANDARDS FOR QUALITY IMPROVEMENT

The 2015 Revision

WFME Office
University of Copenhagen
Denmark
2015

www.wfme.org admin@wfme.org

Explanatory note to guide the use of the Standards

These standards are based on our current understanding of fundamental principles and best practices in designing, maintaining, and enhancing medical education programmes.

Standards are intended to guide medical education programme development and evaluation, facilitate diagnosis of strengths and weaknesses relating to the medical education programme, and to stimulate quality improvement.

Each institution or regulator should review the relevant standards and develop a version of them that is appropriate to the local context. It would be helpful if those local, contextual standards are mapped on to the original WFME standards.

Not all standards may have application in every setting.

A medical school may well receive a satisfactory overall evaluation and maintain accreditation (where appropriate) without necessarily meeting every standard and sub-standard.

Note: In 2017 the document design was updated. The content remains unchanged.

Preface 5

Introduction 6
HISTORY 6
FUNDAMENTALS OF POSTGRADUATE MEDICAL EDUCATION (PME) 8
VALUE OF GLOBAL STANDARDS 9
PREMISES FOR STANDARDS IN POSTGRADUATE MEDICAL EDUCATION 10
USE OF STANDARDS 11

Process And Principles of Revision 13

The WFME Global Standards for Quality Improvement of Postgraduate Medical Education 15
DEFINITIONS 15
THE STANDARDS 16

1. Mission And Outcomes 17
1.1. MISSION 17
1.2. PROFESSIONALISM AND PROFESSIONAL AUTONOMY 19
1.3. EDUCATIONAL OUTCOMES 20
1.4. PARTICIPATION IN FORMULATION OF MISSION AND OUTCOMES 21

2. Educational Programme 22
2.1. FRAMEWORK OF THE PME PROGRAMME 22
2.2. SCIENTIFIC METHOD 23
2.3. PROGRAMME CONTENT 23
2.4. PROGRAMME STRUCTURE, COMPOSITION AND DURATION 24
2.5. ORGANISATION OF EDUCATION 25
2.6. THE RELATION BETWEEN PME AND SERVICE 26

3. Assessment of Trainees 27
3.1. ASSESSMENT METHODS 27
3.2. RELATION BETWEEN ASSESSMENT AND LEARNING 28

4. Trainees 29
4.1. ADMISSION POLICY AND SELECTION 29
4.2. NUMBER OF TRAINEES 30
4.3. TRAINEE COUNSELLING AND SUPPORT 31
4.4. TRAINEE REPRESENTATION 32
4.5. WORKING CONDITIONS 32

5. Trainers 34
5.1. RECRUITMENT AND SELECTION POLICY 34
5.2. TRAINER OBLIGATIONS AND TRAINER DEVELOPMENT 35

6. Educational Resources 36
6.1. PHYSICAL FACILITIES 36
6.2. LEARNING SETTINGS 36
6.3. INFORMATION TECHNOLOGY 37
6.4. CLINICAL TEAMS 38
6.5. MEDICAL RESEARCH AND SCHOLARSHIP 38
6.6. EDUCATIONAL EXPERTISE 39
6.7. LEARNING IN ALTERNATIVE SETTINGS 40

7. Programme Evaluation 41
7.1. MECHANISMS FOR PROGRAMME MONITORING AND EVALUATION 41

7.2. TRAINER AND TRAINEE FEEDBACK 42
7.3. PERFORMANCE OF QUALIFIED DOCTORS 43
7.4. INVOLVEMENT OF STAKEHOLDERS 43

8. Governance and Administration 45

8.1. GOVERNANCE 45
8.2. ACADEMIC LEADERSHIP 45
8.3. EDUCATIONAL BUDGET AND RESOURCE ALLOCATION 46
8.4. ADMINISTRATION AND MANAGEMENT 46
8.5. REQUIREMENTS AND REGULATIONS 47

9. Continuous Renewal 48

Bibliography 50

PREVIOUS AND PRESENT WFME STANDARDS 50
GENERAL 51
PME-RELATED 54

Appendix: Members of Task Force 55

WORKING PARTY 55
INTERNATIONAL PANEL OF EXPERTS 56

WFME Executive Council 61

Preface

Starting in 1998, the World Federation for Medical Education (WFME) developed the WFME Trilogy: *Global Standards for Quality Improvement of Medical Education*, covering all three phases of medical education: *Basic (Undergraduate) Medical Education (BME); Postgraduate Medical Education (PME);* and *Continuing Professional Development (CPD) of Medical Doctors*. Preliminary results were presented in 2000 and the Trilogy was published in 2003.

The global standards for medical education have been implemented and used extensively all over the world. They offer medical education institutions and programmes at various stages of development, and with different educational, socio-economic and cultural conditions and different disease patterns, a template for defining institutional, national and regional standards, and a lever for reform programmes.

As a result, valuable experience was gained and fruitful advice and recommendations compiled from the use of the standards in relation to institutional and national medical education reforms, evaluation and accreditation procedures.

From the outset it was decided that the global standards should not be changed too frequently with the risk of creating unnecessary inconvenience among user institutions. However, ten years after publication of the global standards for medical education, the Executive Council of WFME realised the need for a revision taking into account the commentaries received from medical educators, institutions and organisations and the accumulated relevant literature in the field. Therefore, in 2012 the Federation initiated a revision of the Trilogy.

During the revision process, which involved a small working party and a broad international panel of experts, extensive comments and proposals were received, showing a need for a thorough overhaul. However, it should be emphasised that this revised version of the WFME standards for postgraduate medical education respects the original overall principles and structure. The revised version presents much the same standards at two levels of attainment, basic and developmental, as the original 2003 standards document.

It should be noted that this document concerns professional medical education, not training for academic degrees like PhD and Master.

WFME is profoundly indebted to all who have contributed to the process of reviewing the global standards. The enthusiasm and readiness to assist encountered in all regions have been overwhelming, thereby signalling that the standards are both desirable and feasible.

The WFME Executive Council trusts that the revised standards document will be useful for everybody involved in postgraduate medical education. The document will be of interest for health authorities, medical associations, medical schools and national and international institutions/organisations dealing with postgraduate medical education in all countries. WFME would advise that trustworthy authorities are established country-wise to oversee the application of the global PME standards.

Introduction

HISTORY

The improved health of all peoples is the main goal of medical education. This is also the overall mission of the World Federation for Medical Education (WFME), the international body representing all medical teaching institutions, medical teachers and students and medical doctors in all aspects of their education. In keeping with its constitution, WFME undertakes to promote the highest scientific and ethical standards in medical education, and encourage development of learning methods, new instructional tools, and innovative management of medical education.

Since 1984, WFME has conducted an "International Collaborative Programme for the Reorientation of Medical Education". Cornerstones in this process were the Edinburgh Declaration, 1988, which was adopted by the World Health Assembly, WHA Resolution 42.38, 1989, and the Recommendations of the World Summit on Medical Education, 1993, reflected in WHA Resolution 48.8, Reorientation of Medical Education and Medical Practice for Health for All, 1995.

In compliance with its mandate, WFME launched the programme on global standards in Medical Education in a position paper of 1998. The purpose was to provide a tool for quality improvement in medical education, in a global context, to be applied by institutions responsible for medical education, and in programmes throughout the continuum of medical education.

The WFME programme on global standards in medical education, approved by the World Health Organization (WHO) and the World Medical Association (WMA), had from the very outset three main intentions:

- to stimulate authorities, organisations and institutions having responsibility for medical education to formulate their own plans for change and for quality improvement in accordance with international recommendations;
- to establish a system of national and/or international evaluation, accreditation and recognition of medical educational institutions and programmes to assure minimum quality standards for the programmes; and
- to safeguard practice in medicine and medical manpower utilisation, in the context of increasing internationalisation, by well-defined international standards in medical education.

The WFME global standards embrace all phases of medical education, i.e. basic (undergraduate) medical education, postgraduate medical education and continuing professional development of medical doctors. The trilogy of global standards intends to facilitate the relationship between the phases of medical education.

In developing the Trilogy, WFME appointed three International Task Forces, each comprising a Working Party meeting on a retreat basis, and a broader Panel of Experts that communicated mainly electronically. Members of the Task Forces were selected on basis of their expertise and with geographical coverage an important consideration. The drafts of the standards documents were discussed frequently and in numerous settings around the world. The many commentaries received were collated and taken into account.

Implementation around the world of the standards programme started immediately after the first presentation in 2000 and the conduct of pilot studies in all 6 WFME Regions. The process of implementation was accelerated after broad international endorsement of the standards at the WFME World Conference *Global Standards in Medical Education for Better Health Care* in Copenhagen 2003.

The global standards, translated into several languages, have been used and have influenced national planning of medical education in many countries.

In the early stages of developing global standards for medical education, it became clear that specifying global standards in any restricted sense would exert insufficient impact and would have the potential to lower the quality of medical education. The criticism, whether justified or not, has become commonplace that medical education has adjusted slowly and inadequately both to changing conditions in the health care delivery systems, and to the needs and expectations of societies. Thus, a lever for change and reform was incorporated into the standards. This led the WFME standards to being framed to specify attainment at two different levels: (a) basic standards or minimum requirements; and (b) standards for quality development.

That the WFME standards would have the status of an accreditation instrument was considered from the inception. After deliberation, WFME has taken the position that only nationally appointed agencies can be directly responsible for accreditation procedures. However, WFME can have a role in assisting in accreditation processes and globally adopted standards can function as a template for the agencies designated to implement evaluation and accreditation. WFME, in collaboration with WHO, developed guidelines and procedures for accreditation as an activity within the WHO/WFME Partnership of 2004 to improve medical education.

The medical workforce is globally mobile and the WFME standards have a part to play in safeguarding adequate educational grounding of migrating doctors. However, incentives for retaining locally trained doctors in their own countries and regions are equally essential. The WFME Standards should not be viewed as encouraging increasing medical mobility and spurring brain drain of doctors from developing countries. The world is characterised by increasing internationalisation, from which the medical workforce is not immune, and compliance with the standards should serve as necessary quality-assuring credentials of medical doctors wherever they are based.

To ensure that the competencies of medical doctors are globally applicable and transferable, readily accessible and transparent documentation of the levels of quality of educational institutions and their programmes is essential. The *Avicenna Directory of Medical Schools,* developed by WFME from 2007 to replace the *WHO World Directory of Medical Schools,* aimed to constitute a roster of medical educational institutions, indicating specifically whether institutions included have attained globally approved standards for medical education programmes. The *New World Directory of Medical Schools*, established in 2012 as a merger of the Avicenna Directory and the International Medical Education Directory (IMED) of the Foundation for the Advancement of International Medical Education and Research (FAIMER) has continued this line, and could be an ambitious model for creation of a similar register of postgraduate medical education programmes.

FUNDAMENTALS OF POSTGRADUATE MEDICAL EDUCATION (PME)

Postgraduate medical education is the phase of medical education in which doctors develop competencies under supervision after completion of their basic medical qualification and is usually conducted according to specified regulations and rules. Postgraduate medical education is the final preparation step for certification and/or licensure of doctors. Postgraduate medical training is often used synonymously with postgraduate medical education, but training is in this document considered as a part of postgraduate medical education with special emphasis on practical training such as clinical and communication skills training.

The concept of postgraduate medical education encompasses – depending on the educational programme – the following types of education:

- pre-registration education
- vocational/professional education
- specialist/ subspecialist education
- other formalised education programmes for defined expert functions.

Accomplished pre-registration education is the basis for assigning to doctors the right to independent practice. In some countries such an authorisation is required before specialist/subspecialist education can begin, in other countries right to independent practice is a part of authorisation as specialist or subspecialist. Education for specialisation comprises a number of educational programmes corresponding to the recognized specialities; the number and designations vary between countries and sometimes within a specific country. Education for defined expert functions normally follows some guidelines, but the requirements are normally more flexible than specialist/subspecialist programmes.

In addition to the practical clinical aspects, further theoretical education is required in postgraduate medical education. It should be noted that the present standards for postgraduate medical education do not embrace PhD education programmes.

Postgraduate medical education has developed from a setting similar to apprenticeship, meaning that the young doctors work in e.g. clinical settings together with more experienced colleagues who take the responsibility for their instruction and supervision. Postgraduate medical education in a given speciality ends with the attainment of specialist qualifications which entitles the practitioner to undertake unsupervised practice in that speciality.

Organisation and responsibility for postgraduate medical education programmes varies considerably from country to country, and sometimes also within a country. Authorities, institutions or bodies involved in regulation and management of postgraduate medical education can be national governmental agencies, national or regional boards, universities, medical schools or colleges, medical societies, hospitals or hospital systems, employers, competent professional organisations or a combination of such providers with shared responsibility. Analogous to the situation in basic medical education postgraduate medical education in some countries is the total responsibility of a medical school or a special institution, but frequently responsibility for postgraduate medical education is more complex. For example, the mission and outcomes could be defined by a medical society, whereas the training is often delivered by a hospital system and assessment of trainees undertaken by a third party.

In postgraduate medical education highly sophisticated learning programmes have been developed in some countries, the components of which are planned clinical/practical placements, expert supervision, theoretical teaching, research experience, systematic assessments and evaluation of the education programmes. In other countries, the process of postgraduate medical education remains more traditional based almost exclusively on practical clinical training under supervision.

Internationally, there has over the last decades been a trend in some areas of the world to introduce supranational regulations of postgraduate medical education. Consensus about standards for medical education might facilitate convergence of the development of postgraduate medical education.

VALUE OF GLOBAL STANDARDS

A central part of the WFME strategy is to develop global standards and guidelines for medical education, that are supportive of the institutions concerned, their educational programmes, the medical profession, and the individual student and doctor. These global standards constitute a framework, serving as a yardstick against which those responsible for postgraduate medical education can evaluate their own activities and organisations. Moreover, globally accepted standards could be used as a basis for national and regional approval and accreditation of educational programmes.

Equally relevant for global standards is the process of medical education. Desirable practices in educating the basic doctor, incorporating well-recognised and accepted principles of learning, together with the institutional conditions for educational activities, must form the basis for global standards.

Moreover, quality assurance of medical education must emphasise the need for improvement and provide guidance for achieving it. This will avoid interpretation of standards as a levelling at a lower level of quality among institutions.

Standards are not an »either/or« matter, but a matter of specific conduct and intentional planning. Furthermore, some postgraduate medical programmes might develop so unique a quality as to go beyond standards achieved by most programmes. Such qualities might, in the long run, serve as examples for new goal-settings.

Standards must be clearly defined, and be meaningful, appropriate, relevant, measurable, achievable and accepted by the users. They must have implications for practice, acknowledge diversity and foster adequate development.

Evaluation based on generally accepted standards is an important incentive for improvement and for raising the quality of medical education, both when reorientation and reform are pursued, and also when continuous development is strived for.

WFME considers that the operation of standards can promote discussion and stimulate development of consensus about objectives, and will help schools to formulate essentials of their educational programmes and to describe the fundamentals of medical education. Standards will

broaden opportunities for educational research and development and foster discussion and cooperation across departmental and other boundaries.

The existence of standards will empower educators in their effort to bring about change, and will serve to guide trainees in their choices.

For curriculum planners, acceptance of standards will save time and resources. Adoption of standards for evaluation will provide valuable information for providers of funds, politicians and society.

Placing medical education on a basis of shared global standards will facilitate exchange of medical students, and ease the acceptance of medical doctors in countries other than those in which they trained. In consequence, it will diminish the burden of judging the competencies of doctors who have been educated in medical schools in different countries.

Finally, low quality programmes can be improved by use of a system of evaluation and accreditation based on internationally accepted standards. This is likely to enhance the quality of health care, both nationally and internationally.

PREMISES FOR STANDARDS IN POSTGRADUATE MEDICAL EDUCATION

The Executive Council of WFME is evidently of the view that global standards for medical education, which have general applicability, can be defined. These definitions take account of the variations in the process, structure, content, outcomes/competencies, assessment and learning environment of medical education among countries, due to differences in teaching traditions, culture, socio-economic conditions, the health and disease spectrum, and the different forms of health care delivery systems. Similar differences can also occur within individual countries. Nevertheless, the scientific basis of medicine and the necessity to base clinical practice on evidence is universal; the task of medical education everywhere, throughout its phases, is the provision of high quality health care. Notwithstanding great diversity, there is an increasing degree of convergence of structure, process and product of medical education worldwide.

Global standards in postgraduate medical education, as for other phases of medical education, must be specified, modified or supplemented in accordance with regional, national and institutional needs and priorities. WFME stresses that there can be no benefit in fostering uniformity of educational programmes and learning activities and hereby jeopardising social accountability. Moreover, quality assurance of medical training programmes must give emphasis to improvement, and provide guidance for advancement, instead of simply advocating “fulfilment of standards” as the ultimate goal. It is the prerogative of any national accrediting body to determine the level that will be examined for evaluation/accreditation.

In drafting standards for postgraduate medical education, the WFME applied the principles which were used in developing the global standards for basic medical education. Attention was given to the application of general guidelines in quality development of medical education. Therefore, for global standards in postgraduate medical education to be generally accepted, the following premises were adopted:

- Only general aspects of postgraduate medical education and training should be covered.
- Standards should be concerned with broad categories of process, structure, content, outcomes/competencies, assessment and learning environment.
- Standards should function as a lever for change and reform.
- Standards are intended not only to set minimum global requirements but also to encourage quality development beyond the levels specified.
- Standards should be formulated in such a way that they acknowledge regional and national differences in the educational programme, and allow for different local, national and regional profiles and developments.
- Compliance with standards must be a matter for each community, country or region.
- Use of a common set of global standards does not imply or require equivalence of programme content and outcomes of postgraduate medical education, but deviations should be clearly described and motivated.
- Standards should acknowledge the dynamic nature of programme development.
- Standards should be formulated as a tool which authorities, organisations and institutions responsible for postgraduate medical education can use as a model for their own programme development.
- Standards should not be used to rank education programmes.
- Standards should be further developed through broad international discussion and consensus.
- The value of the standards must be tested in evaluation studies in each region.
- Standards must be clearly defined, and be meaningful, appropriate, relevant, measurable, achievable and accepted by the users. They must have implications for practice, recognise diversity and foster adequate development.
- Standards must be formulated in collaboration with stakeholders.

USE OF STANDARDS

It should be emphasised that, in working with the standards for purposes of programme development or evaluation, the principles underlying each standard are the essential points. Over-attention to details should not obscure the need to apply the *basic standards*, and the desirability of working towards the *standards for quality development*. WFME wants to stress that all details in the standards document should not necessarily be fulfilled by every postgraduate medical education programme.

WFME holds that the set of standards, offering as it does a developmental perspective from attainment of basic to quality development levels, can be used globally as a tool for quality assurance and development of postgraduate medical education in the following ways:

- **Self – evaluation of programmes**

  The primary intention of the WFME in introducing an instrument for quality improvement is to provide a new framework against which authorities, organisations and institutions with responsibility for postgraduate medical education can measure themselves in voluntary self-evaluation and self-improvement processes. The standards can thus be considered a self-study manual.

- ***External evaluation or peer review***

  The process of institutional self-evaluation described can be further enhanced and objectivity promoted by inclusion of evaluation and counselling from external peer review committees.

- ***Combination of self-evaluation and external evaluation or peer review***

  The WFME considers such a combination to be the most valuable method.

- ***Approval and accreditation***

  Depending on local needs and traditions, the standards can also be used by national or regional agencies dealing with approval

Process And Principles of Revision

It was decided that the WFME standards should remain formulated as a combination of process, structure, content, outcomes/competencies assessment and learning environment standards.

The plan used for the 2015 revision of the postgraduate medical education standards document was:

Phase I:
Production of a draft by a small working party of persons associated with the WFME office.

Phase II:
Gathering of comments and proposals for amendments and additions from a broad international panel of experts representing all six WFME Regions.

Phase III:
Presentation of the amended document for further comments from the main partners of WFME, including the members of the WFME Executive Council.

In this work the original premises for standards in postgraduate medical education were followed, but it was realised that a system should be introduced to allow clearer presentation of standards. Annotations should provide clarification and exemplification based on accumulated experiences in using the standards. Formulations should be harmonised between the three sets of WFME global standards.

General principles underpinning the standards were not changed during the revision, e.g. using two levels of attainment, i.e. basic standards and standards for quality development, and not only minimum requirements. The dividing line between basic standards and standards for quality development was considered and changed in some cases in accordance with developments in requirements to medical education. The number of areas and subareas is basically the same, but composite standards have been split to increase the overview of standards content. The sequence of standards is in some cases changed. A numbering system has been introduced, thereby facilitating references to and communication about standards.

As in the standards for basic medical education it was regarded desirable to clearly place and define the responsibility for fulfilment of the standards. In this document, we have chosen the concept of the **programme provider or a group of providers** with shared responsibility as the overall responsible actor(s). Explicit responsibility for action to achieve all standards is now placed with the programme provider(s).

The classification of standards has been kept close to the original, but minor reformulation of some areas and standards was needed to clarify the content. Repetition and overlap have been reduced. Many standards specify that implementation rather than just the simple formulation of the policy is required.

The revised set of WFME standards appears to be somewhat more detailed. However, this is more a result of editorial revision.

To aid better understanding, the number of annotations was significantly expanded. The intention is to clarify the meaning of the standards, primarily by explanation and exemplification of e.g. activities and conditions, taking into account social and cultural differences. This should also facilitate the planning of data collection for self-evaluation studies and external evaluations and provide a more secure basis for translations

The WFME Global Standards for Quality Improvement of Postgraduate Medical Education

DEFINITIONS

Postgraduate medical education may be defined as the phase in which doctors develop competencies under supervision towards independent practice after completion of their basic medical qualification, and might comprise pre-registration education (leading to right to independent practice), systematic vocational/professional education, specialist and sub-specialist education or other formalised education programmes for defined expert functions. Upon completion of a formal postgraduate training programme a degree, diploma or certificate is usually granted (cf. fundamentals of postgraduate medical education).

It should be emphasised that life-long learning is essential in medicine, which means that completed postgraduate medical education must be followed by the process of continuing professional development.

In the standards document the term **programme provider(s)** refer to local and national authorities or bodies involved in regulation and management of postgraduate medical education, and could be a national governmental agency, a national or regional board, a university, a college, a medical society, a hospital or hospital system, a competent professional organisation or a combination of such providers with shared responsibility.

The WFME recommends the following set of global standards for postgraduate medical education. The set of standards is structured according to **9 areas** with a total of **36 sub-areas**, being aware of the complex interactions and links between them.

AREAS are defined as the broad components to be covered in the process, structure, content, outcomes/competencies, assessment and learning environment of postgraduate medical education and training. They are:

1. Mission and outcomes
2. Educational programme
3. Assessment of trainees
4. Trainees
5. Trainers
6. Educational resources
7. Programme evaluation
8. Governance and administration
9. Continuous renewal

SUB-AREAS are defined as specific aspects or dimensions of an area, corresponding to performance indicators.

STANDARDS are specified for each sub-area using two levels of attainment:

Basic standard.
This means that the standard must be met and fulfilment demonstrated during evaluation of the education programme.

Basic standards are expressed by a "**must**".

Standard for quality development.

This means that the standard is in accordance with international consensus about best practice for postgraduate medical education. Fulfilment of – or initiatives to fulfill - some or all of such standards should be documented. Fulfilment of these standards will vary with the stage and development of the education programme, available resources, the educational policy and other local conditions influencing relevance, priorities and possibilities. Even the most advanced programmes might not comply with all standards.

Standards for quality development are expressed by a "**should**".

ANNOTATIONS are used to clarify, amplify or exemplify formulations of the standards. No new requirements are introduced in the annotations.

The listing of examples in annotations are in some cases exhaustive, in others not. It should also be noted, that a postgraduate medical education programme will rarely use and possess all the characteristics mentioned in examples.

THE STANDARDS

The 2015 revision of the WFME global standards for quality improvement of postgraduate medical education, comprising altogether 161 basic standards, 94 quality development standards and 123 annotations, are presented in the following section.

1. Mission And Outcomes

1.1. MISSION

Basic standards:
The programme provider(s) **must**

- state the mission of the programme. (B 1.1.1)
- make the mission publicly known to the health sector it serves. (B 1.1.2)
- base the mission on
  - consideration of the health needs of the community or society. (B 1.1.3)
  - the needs of the health care delivery system. (B 1.1.4)
  - other aspects of social accountability, as appropriate. (B 1.1.5)
- outline the programme containing both theoretical and practice-based components, with emphasis on the latter, resulting in a medical doctor who is
  - competent to undertake comprehensive appropriate medical practice in the defined field of medicine. (B 1.1.6)
  - capable of working in a professional manner. (B 1.1.7)
  - able to work unsupervised and independently. (B 1.1.8)
  - able to work within a professional/interprofessional team when relevant. (B 1.1.9)
  - committed and prepared to life-long learning and participation in continuing medical education/continuing professional development. (B 1.1.10)
- ensure improvement of patient care that is appropriate, effective, compassionate and safe in dealing with health problems and promotion of health, including a patient-centred and holistic approach. (B 1.1.11)
- ensure that trainees have appropriate working conditions to maintain their own health. (B 1.1.12)

Quality development standards:
The programme provider(s) **should** encourage

- appropriate innovation in the education process allowing for development of broader and more specialised competencies than those identified within the basic required competencies. (Q 1.1.1)
- doctors to become scholars within their chosen field of medicine. (Q 1.1.2)
- doctors to become active participants in facing social determinants of health. (Q 1.1.3)

Annotations:

- *Mission* provides the overarching frame to which all other aspects of the programme must be related. The mission statement would include general and specific issues relevant to institutional, national, regional and, if relevant, global policy and health needs. Mission in this document includes visions about postgraduate medical education.

- *The programme provider(s)* would include local and national authorities or bodies involved in regulation and management of postgraduate medical education, and could be a national governmental agency, a national or regional board, a university, a college, a medical society, a hospital or hospital system, a competent professional organisation or a combination of such providers with shared responsibility.
- *Make the mission publicly known* means to make it known to the health sector as well as the general public.
- The *health sector* would include the health care delivery system, whether public or private, and medical research institutions.
- Encompassing the *health needs of the community* would imply interaction with the local community, especially the health and health related sectors, and adjustment of the programme to demonstrate attention to and knowledge about health problems of the community.
- *Social accountability* would include willingness and ability to respond to the needs of society, of patients and the health and health related sectors and to contribute to the national and international development of medicine by fostering competencies in health care, medical education and medical research.

  Social accountability is sometimes used synonymously with social responsibility and social responsiveness. In matters outside the control of the programme provider, it would still be possible to demonstrate social accountability through advocacy and by explaining relationships and drawing attention to consequences of the policy.
- *Life-long learning* is the professional responsibility to keep up to date in knowledge and skills through appraisal, audit, reflection or recognised continuing professional development (CPD)/continuing medical education (CME) activities.
- *Continuing medical education (CME)* refers to life-long continuing education in the knowledge, skills and attitudes of medical practice.
- *Continuing professional development (CPD)* refers to life-long professional activities that doctors undertake, formally and informally, to maintain, update, develop and enhance their knowledge, skills and attitudes in response to the needs of their patients and their own personal development. CPD is a broader concept than CME.
- *Compassionate care* would include awareness of patient and family aspects of matters related to the end of life.
- *Trainees* refer to doctors in postgraduate education.
- *Scholar* refers to an individual with deeper and/or broader engagement in the advancement of the discipline, including participation in academic development and advanced education and research in medicine.
- *Chosen field of medicine* would include recognised specialties, including general practice, subspecialties and expert functions. The formulation of the standards recognise that the number, designations and content of specialties, subspecialties and expert areas vary significantly from country to country.

1.2. PROFESSIONALISM AND PROFESSIONAL AUTONOMY

Basic standards:
The programme provider(s) **must**

- include professionalism in the education of doctors. (B 1.2.1)
- foster the professional autonomy necessary to enable the doctor to act in the best interests of the patient and the community. (B 1.2.2)

Quality development standards:
The programme provider(s) **should**

- ensure a collaborative relationship with government and other counterparts, whilst maintaining appropriate independence from them. (Q 1.2.1)
- ensure academic freedom. (Q 1.2.2)

Annotations:

- *Professionalism* describes the knowledge, skills, attitudes and behaviours expected by patients and community from individual doctors during the practice of their medical profession and includes skills of lifelong learning and maintenance of competencies, information literacy, ethical behaviour, integrity, honesty, altruism, empathy, service to others, adherence to professional codes, justice and respect for others, including consideration of patient safety. The perception of professionalism should reflect any ethical guidance produced by the national medical regulator.
- *Autonomy* in the patient-doctor relationship would ensure that doctors at all times make informed decisions in the best interest of their patients and the society, based on the best available evidence. Autonomy related to doctors' learning implies that they have some influence on decisions about what to learn and how to plan and carry out learning activities. It also implies access to the knowledge and skills doctors need to keep abreast in meeting the needs of their patients and the society, and that the sources of knowledge are independent and unbiased. In acting autonomously, possible guidelines should be taken into consideration.
- *Other counterparts* would include regional and local authorities outside the education system, cultural and religious groupings, private companies, unions and other interest groups who might influence the provider to make decisions about key areas such as design of the programme (cf. 2.1 and 2.4), assessments (cf. 3.1), trainee recruitment (cf. 4.1 and 4.2), trainer recruitment/selection (cf. 5.1) and employment conditions and resource allocation (cf. 8.3).
- *Appropriate independence* will have to be defined according to principles for national regulations.
- *Academic freedom* would include appropriate freedom of expression, freedom of inquiry and publication.

1.3. EDUCATIONAL OUTCOMES

Basic standards:
The programme provider(s) **must**

- define the intended educational outcomes of the programme with respect to
  - achievements at a postgraduate level regarding knowledge, skills and attitudes. (B 1.3.1)
  - appropriate foundation for the future career of trainees in the chosen field of medicine. (B 1.3.2)
  - future roles in the health sector. (B 1.3.3)
  - commitment to and skills in life-long learning. (B 1.3.4)
  - the health needs of the community, the needs of the health care system and other aspects of social accountability. (B 1.3.5)
  - professional behaviour. (B 1.3.6)
  - generic and discipline/speciality-specific components. (B 1.3.7)
  - appropriate conduct regarding patients and their relatives, fellow trainees, trainers and other health care personnel. (B 1.3.8)
- ensure appropriate trainee conduct with respect to colleagues and other health care personnel, patients and their relatives. (B 1.3.9)
- make the intended outcomes publicly known. (B 1.3.10)

Quality development standards:
The programme provider(s) **should**

- ensure interaction between basic and postgraduate medical education. (Q 1.3.1)

Annotations:

- *Educational outcomes* or learning outcomes/competencies refer to statements of knowledge, skills and attitudes that trainees demonstrate at the end of a period of learning, the educational results. Outcomes might be either intended outcomes or acquired outcomes. Intended outcomes are often used for formulation of educational/learning objectives. Outcomes include competencies.
  Outcomes within medicine and medical practice – to be specified by the responsible authority – would include documented knowledge and understanding of relevant (a) basic biomedical sciences, (b) behavioural and social sciences, (c) medical ethics, human rights and medical jurisprudence relevant to the practice of medicine, and (d) clinical sciences, including clinical skills with respect to diagnostic procedures, practical procedures, communication skills, treatment (including palliative care) and prevention of disease, health promotion, rehabilitation, clinical reasoning and problem solving. It also includes skills in doctor-patient relationship with emphasis on a compassionate attitude and humanity.

The characteristics and achievements the trainee would display upon completion of the programme might be categorised in terms of the roles of the doctor. Such roles would be (a) medical practitioner or medical expert, (b) communicator, (c) collaborator/team worker, (d) leader/manager or administrator, (e) health advocate, (f) scholar and scientist contributing to development and research in the chosen field of medicine, (g) teacher, supervisor and trainer to colleagues, medical students and other health professions and (h) a professional. Similar frameworks could be defined.

- *Generic components* would include all general aspects of medicine relevant for the function of the doctor.
- *Discipline/speciality specific components* refer to the knowledge, skills and attitudes of the chosen field of medicine as a speciality, subspeciality or expert function.
- *Appropriate conduct* could presuppose a written code of professsional and personal conduct.
- *Basic medical education* refers to the basic (undergraduate) programmes in medicine conducted by medical schools/medical faculties/ medical colleges or medical academies leading to outcomes at a basic level.

1.4. PARTICIPATION IN FORMULATION OF MISSION AND OUTCOMES

Basic standards:
The programme provider(s) **must**

- state the mission and define the intended educational outcomes of the programmes in collaboration with principal stakeholders. (B 1.4.1)

Quality development standards:
The programme provider(s) **should**

- base the formulation of mission and intended educational outcomes of the programmes on input from other stakeholders. (Q 1.4.1)

Annotations:

- *Principal stakeholders* would include trainees, programme directors, medical scientific societies, hospital administrations, governmental authorities, other health care authorities and professional associations or organisations as well as representatives of supervisors, trainers and teachers. Some principal stakeholders may be programme providers as well.
- *Other stakeholders* would include representatives of other health professions, patients, the community and public (e.g. users of the health care delivery systems, including patient organisations). Other stakeholders would also include other representatives of academic and administrative staff, medical schools, education and health care authorities, professional organisations and medical scientific societies.

2. Educational Programme

2.1. FRAMEWORK OF THE PME PROGRAMME

Basic standards:
The programme provider(s) **must**

- determine the educational framework based upon the intended educational outcomes of the programme and the qualifications of the trainees. (B 2.1.1)
- build its educational framework on the acquired outcomes of existing basic medical education. (B 2.1.2)
- organise the educational framework in a systematic and transparent way. (B 2.1.3)
- use practice-based training involving the personal participation of the trainee in the services and responsibilities of patient care. (B 2.1.4)
- use instructional and learning methods that are appropriate and ensure integration of practical and theoretical components. (B 2.1.5)
- deliver the programme in accordance with principles of equality. (B 2.1.6)
- use a trainee-centred approach that stimulates, prepares and supports trainees to take responsibility for their own learning process and to reflect on their own practice. (B 2.1.7)
- guide the trainee by means of supervision and regular appraisal and feedback. (B 2.1.8)
- inform trainees about the programme and the rights and obligations of trainees. (B 2.1.9)
- include the commitment to ethical considerations in the programme. (B 2.1.10)

Quality development standards:
The programme provider(s) **should**

- increase the degree of independent responsibility of the trainee as skills, knowledge and experience grow. (Q 2.1.1)
- recognise gender, cultural and religious specifications and prepare the trainee to interact appropriately. (Q 2.1.2)

Annotations:

- *Framework of the programme* in this document refers to specification of the educational programme, including a statement of the intended educational outcomes (cf. 1.3), the content/syllabus, experiences and processes of the programme (cf. 2.2-2.5). Also, the framework would include a description of the planned instructional and learning methods and assessment methods (cf. 3.1).
- *Instructional and learning methods* would encompass any didactic, participatory demonstration or supervised teaching and learning methods such as lectures, small-group teaching, problem-based or case-based learning, peer-assisted learning, practicals, laboratory exercises, bed-side teaching, clinical demonstrations, clinical skills laboratory training, field exercises in the community, web-based instructions and not least practical clinical work as a junior member of the staff.

- *Integration of practical and theoretical components* can take place in didactic learning sessions and supervised patient care experiences as well as through self-directed and active learning.
- *Delivery in accordance with principles of equality* means equal treatment of staff and trainees irrespective of gender, ethnicity, religion, political affiliation, sexual orientation or socio-economic status, and taking into account physical capabilities.

2.2. SCIENTIFIC METHOD

Basic standards:
The programme provider(s) **must**

- introduce in the programme the foundation and methodology of medical research, including clinical research and clinical epidemiology. (B 2.2.1)
- ensure that the trainee
  - becomes able to use scientific reasoning. (B 2.2.2)
  - becomes familiar with evidence-based medicine through exposure to a broad range of relevant clinical/practical experience in different settings in the chosen field of medicine. (B 2.2.3)

Quality development standards:
The programme provider(s) **should**

- include formal teaching on critical appraisal of the literature and scientific data. (Q 2.2.1)
- adjust the content to scientific developments. (Q 2.2.2)

Annotations:

- *Evidence-based medicine* means medicine founded on documentation, trials and accepted scientific results.

2.3. PROGRAMME CONTENT

Basic standards:
The programme provider(s) **must**

- include in the programme clinical work and relevant theory or experience of
  - basic biomedical, clinical, behavioural and social sciences and preventive medicine. (B 2.3.1)
  - clinical decision-making. (B 2.3.2)
  - communication skills. (B 2.3.3)
  - medical ethics. (B 2.3.4)
  - public health. (B 2.3.5)
  - medical jurisprudence and forensic medicine. (B 2.3.6)
  - managerial disciplines. (B 2.3.7)

- patient safety. (B 2.3.8)
- doctors' self-care. (B 2.3.9)
- the interface with complementary medicine. (B 2.3.10)
- organise the programme with appropriate attention to patient safety and autonomy. (B 2.3.11)

Quality development standards:
The programme provider(s) **should**

- improve the content regarding of knowledge, skills and attitudes related to the various roles of the doctor. (Q 2.3.1)
- adjust the content to changing contexts and needs of the health care delivery system. (Q 2.3.2)

Annotations:

- *The basic biomedical sciences* would – depending on local needs, interests, traditions and speciality needs - typically include anatomy, biochemistry, biophysics, cell biology, genetics, immunology, microbiology (including bacteriology, parasitology and virology), molecular biology, pathology, pharmacology and physiology.
- *The clinical sciences* would include the chosen clinical or laboratory discipline (medical speciality, subspeciality or expert function) and in addition other relevant clinical/laboratory disciplines.
- *The behavioural and social sciences* would - depending on local needs, interests and traditions - typically include biostatistics, community medicine, epidemiology, global health, hygiene, medical anthropology, medical psychology, medical sociology, public health and social medicine and would provide the knowledge, concepts, methods, skills and attitudes necessary for understanding socio-economic, demographic and socio-cultural determinants of causes, distribution and consequences of health problems.
- *Managerial disciplines* would focus on education in leadership roles, taking into account the need for leadership training to teach trainees how to create change. Also, these disciplines would focus on developing relevant managerial skills in practice, such as e.g. determining priorities or cost-effectiveness of health care and knowledge of referral systems.
- *Complementary medicine* would include unorthodox, traditional or alternative practices.
- *Various roles of the doctor,* cf. 1.3, annotation.

2.4. PROGRAMME STRUCTURE, COMPOSITION AND DURATION

Basic standards:
The programme provider(s) **must**

- describe the overall structure, composition and duration of the programme. (B 2.4.1)
- state compulsory and optional components of the programme. (B 2.4.2)
- integrate practice and theory. (B 2.4.3)
- consider national regulations. (B 2.4.4.)

- provide adequate exposure to how local, national or regional health systems address the health care needs of populations. (B 2.4.5)

Quality development standards:
The programme provider(s) **should**

- in making a decision about the duration of the programme, take into consideration
  - the acquired outcomes of basic medical education related to the chosen field of medicine. (Q 2.4.1)
  - requirements of the different roles of the trained doctor in the health sector. (Q 2.4.2)
  - possible alternatives to the use of time-based definitions of education. (Q 2.4.3)

Annotations:

- *Overall structure* would include the sequence of attachments to the training settings.
- *Integration of practice and theory* would include self-, group- and didactic learning sessions and supervised patient care experiences.
- *Health sector,* see 1.1 annotations.
- *Possible alternatives to the use of time-based definitions of education* would e.g. be outcomes-defined programmes, measurements of competencies, log-books of clinical skills and workplace experiences. Such alternatives depend highly on agreed valid and reliable methods of measuring individual achievements.

2.5. ORGANISATION OF EDUCATION

Basic standards:
The programme provider(s) **must**

- define responsibility and authority for organising, coordinating, managing and evaluating the individual educational setting and process. (B 2.5.1)
- include in the planning of the programme appropriate representation of principal as well as other stakeholders. (B 2.5.2)
- plan the education to expose the trainee to a broad range of experiences in the chosen field of medicine. (B 2.5.3)

Quality development standards:
The programme provider(s) **should**

- ensure multi-site education. (Q 2.5.1)
- coordinate multi-site education to gain adequate exposure to different aspects of the chosen field of medicine. (Q 2.5.2)

Annotations:

- *Principal stakeholders*, cf. 1.4, annotation.
- *Other stakeholders*, cf. 1.4, annotation.
- *Multi-site education* would imply the use of various settings characterized by size, patient categories, degree of specialisation (e.g. primary, secondary and tertiary care), in-patient or out-patient clinics, etc.

2.6. THE RELATION BETWEEN PME AND SERVICE

Basic standards:
The programme provider(s) **must**

- describe and respect the apprenticeship nature of professional development. (B 2.6.1)
- integrate training and service. (B 2.6.2)
- ensure that training is complementary to and integrated with service demands. (B 2.6.3)

Quality development standards:
The programme provider(s) **should**

- effectively organise use of the capacity of the health care system for service based training purposes. (Q 2.6.1)

Annotations:

- *Integrate training and service* means on the one hand delivery of proper health care service by the trainees and on the other hand that learning opportunities are embedded in service functions (on-the-job training).
- *Complementary* means that training and service ought to be jointly planned and organised to enhance each other. This would be expressed in an affiliation agreement between the training providers and the service institutions.
- *Effectively organise* refers to the use of different clinical settings, patients and clinical problems for training purposes, and at the same time respecting service functions.

3. Assessment of Trainees

3.1. ASSESSMENT METHODS

Basic standards:
The programme provider(s) **must**

- formulate and implement a policy of assessment of the trainees. (B 3.1.1)
- define, state and publish the principles, purposes, methods and practices for assessment of trainees, including specialist examinations where used. (B 3.1.2)
- ensure that assessments cover knowledge, skills and attitudes. (B 3.1.3)
- use a complementary set of assessment methods and formats according to their "assessment utility", including use of multiple assessors and multiple assessment methods. (B 3.1.4)
- state the criteria for passing examinations or other types of assessment, including number of allowed retakes. (B 3.1.5)
- evaluate and document the reliability, validity and fairness of assessment methods. (B 3.1.6)
- use a system of appeal of assessment results based on principles of natural justice or due (legal) process. (B 3.1.7)

Quality development standards:
The programme provider(s) **should**

- encourage the use of external examiners. (Q 3.1.1)
- incorporate new assessment methods where appropriate. (Q 3.1.2)
- record the different types and stages of training in a training log-book. (Q 3.1.3)

Annotations:

- *Assessment methods* would include consideration of the balance between formative and summative assessment, the number of examinations and other tests, the balance between different types of examinations (written and oral), the use of normative and criterion-referenced judgements, and the use of personal portfolio and log-books and special types of examinations, e.g. objective structured clinical examinations (OSCE) and mini clinical evaluation exercise (MiniCEX). It would also include systems to detect and prevent plagiarism.
- *Specialist examinations* would be conducted by providers or by separate agencies, e.g. colleges or consortia.
- *"Assessment utility"* is a term combining validity, reliability, educational impact, acceptability and efficiency of the assessment methods and formats in relation to intended educational outcomes.
- *Evaluation and documentation of reliability and validity of assessment methods* would require an appropriate quality assurance process of assessment practices. Evaluation of assessment methods may include an evaluation of how they promote education and learning.
- Use of *external examiners* may increase fairness, quality and transparency of assessments.

3.2. RELATION BETWEEN ASSESSMENT AND LEARNING

Basic standards:
The programme provider(s) **must**

- use assessment principles, methods and practices that
  - are clearly compatible with intended educational outcomes and instructional methods. (B 3.2.1)
  - ensure that the intended educational outcomes are met by the trainees. (B 3.2.2)
  - promote trainee learning. (B 3.2.3)
  - ensure adequacy and relevance of education. (B 3.2.4)
  - ensure timely, specific, constructive and fair feedback to trainees on the basis of assessment results. (B 3.2.5)

Quality development standards:
The programme provider(s) **should**

- use assessment principles, methods and practices that
  - encourage integrated learning. (Q 3.2.1)
  - encourage involvement of practical clinical work. (Q 3.2.2)
  - facilitate interprofessional education. (Q 3.2.3)

Annotations:

- *Assessment principles, methods and practices* refer to the assessment of trainee achievement and would include assessment in all domains: knowledge, skills and attitudes.
- *Encouragement of integrated learning* would include consideration of using integrated assessment, while ensuring reasonable tests of knowledge of individual disciplines or subject areas.

4. Trainees

4.1. ADMISSION POLICY AND SELECTION

Basic standards:
The programme provider(s) **must**

- consider the relationship between the mission of the programme and selection of trainees. (B 4.1.1)
- ensure a balance between the education capacity and the intake of trainees. (B 4.1.2)
- formulate and implement a policy on
  - the criteria and the process for selection of trainees. (B 4.1.3)
  - admission of trainees with disabilities requiring special facilities. (B.4.1.4)
  - transfer of trainees from other national or international programmes. (B.4.1.5)
- ensure a high level in understanding of basic biomedical sciences achieved at the undergraduate level before starting postgraduate education. (B 4.1.6)
- ensure transparency and equity in selection procedures. (B 4.1.7)

Quality development standards:
The programme provider(s) **should**

- consider in its selection procedure specific capabilities of potential trainees in order to enhance the result of the education process in the chosen field of medicine. (Q 4.1.1)
- include a mechanism for appeal against decisions related to admission and continuation. (Q 4.1.2)
- include trainees' organisations and other stakeholders in the formulation of the selection policy and process. (Q 4.1.3)
- periodically review the admission policy. (Q 4.1.4)

Annotations:

- *Admission policy* would imply adherence to possible national regulation as well as adjustment to local circumstances. If the programme provider does not control the admission policy, the provider would demonstrate responsibility by explaining to authorities the relationships and drawing attention to consequences, e.g. imbalance between intake and education capacity.
- *Education capacity* refers to all resources needed to deliver the programme, e.g. number of trainers, patients and facilities.
- *Criteria for selection* may include consideration of balanced intake according to gender, ethnicity and other social requirements (socio-cultural and linguistic characteristics of the population), including the potential need of a special recruitment, admission and induction policy for minorities and doctors from underserved rural communities.
- The *process for selection* of trainees would include both rationale and methods of selection such as medical school results, other academic or educational experiences, entrance examinations and interviews, including evaluation of motivation for education in the chosen field of medicine.

- The *policy for admission of trainees with disabilities* will have to be in accordance with national law and regulations and would take into account consideration of both patient and doctor safety.
- *Transfer of trainees* would include trainees from other types of education programmes.
- *Other stakeholders,* cf. 1.4, annotation.
- *Periodically review the admission policy* would be based on relevant societal and professional data to comply with the health needs of the community and society and would include consideration of intake to gender, ethnicity and other social requirements (socio-cultural and linguistic characteristics of the population), including the potential need of a special recruitment, admission and induction policy for underprivileged trainees. The selection criteria should reflect the capability of trainees to achieve competencies and to cover the variations in required competencies related to the diversity of the chosen field of medicine.

4.2. NUMBER OF TRAINEES

Basic standards:
The programme provider(s) **must**

- set a number of education positions that is proportionate to
  - the clinical/practical training opportunities. (B 4.2.1)
  - the capacity for appropriate supervision. (B 4.2.2)
  - other resources available. (B 4.2.3)
  - available information about the health needs of the community and society. (B 4.2.4)

Quality development standards:
The programme provider(s) **should**

- review the number of trainees through consultation with stakeholders. (Q 4.2.1)
- adapt the number of training positions, taking into account
  - available information about the number of qualified candidates. (Q 4.2.2)
  - available information about the national and international market forces. (Q 4.2.3)
  - the inherent unpredictability of precise physician manpower needs in the various fields of medicine. (Q 4.2.4)

Annotations:

- Decisions on *number of trainees* would imply necessary adjustments to national and regional requirements for medical workforce within the chosen field of medicine. If the programme provider does not control trainee intake, it demonstrates responsibility when explaining relationships and drawing attention to problems, e.g. imbalance between intake and education capacity.

- *The health needs of the community and society* would include consideration of intake according to gender, ethnicity and other socio-cultural and linguistic characteristics of the population, including the potential need of a special recruitment, admission and motivation policy for minorities and rural groups of doctors. Forecasting the health needs of the community and society for trained physicians includes estimation of various market and demographic forces as well as the scientific development and migration patterns of physicians.
- *Stakeholders* would include principal as well as other stakeholders, cf. 1.4, annotation.

4.3. TRAINEE COUNSELLING AND SUPPORT

Basic standards:
The programme provider(s) **must**

- ensure access to a system for academic counselling of trainees. (B 4.3.1)
- base the academic counselling of trainees on monitoring the progress in education including reported unintended incidents. (B 4.3.2)
- make support available to trainees, addressing social, financial and personal needs. (B 4.3.3)
- allocate resources for social and personal support of trainees. (B 4.3.4)
- ensure confidentiality in relation to counselling and support. (B 4.3.5)
- offer career guidance and planning. (B 4.3.6).

Quality development standards:
The programme provider(s) **should**

- provide support in case of a professional crisis. (Q 4.3.1)
- involve trainees' organisations in solving problematic trainee situations. (Q 4.3.2)

Annotations:

- *Academic counselling* would include advice on choice of postgraduate education programme. Organisation of counselling would include appointing academic mentors for individual trainees or small groups of trainees and should be conducted in collaboration with professional medical organisations.
- *Unintended incidents* mean incidents potentially harmful to the patient.
- *Addressing social, financial and personal needs* would mean professional support in relation to social and personal problems and events, housing problems, health problems and financial matters, and would include access to health clinics, immunisation programmes and health/disability insurance as well as financial aid services in forms of bursaries, scholarships and loans.
- *Professional crisis* would e.g. be the result of involvement in malpractice or fundamental disagreement with supervisors or colleagues.

4.4. TRAINEE REPRESENTATION

Basic standards:
The programme provider(s) **must**

- formulate and implement a policy on trainee representation and appropriate participation in the
  - statement of mission and intended educational outcomes. (B 4.4.1)
  - design of the programme. (B 4.4.2)
  - planning of trainees' working conditions. (B 4.4.3)
  - evaluation of the programme. (B 4.4.4)
  - management of the programme. (B 4.4.5)

Quality development standards:
The programme provider(s) **should**

- encourage trainees' organisations to be involved in decisions about education processes, conditions and regulations. (Q 4.4.1)

Annotations:

- *Trainee representation* would include participation in groups or committees responsible for programme planning and implementation at the local or national level.

4.5. WORKING CONDITIONS

Basic standards:
The programme provider(s) **must**

- carry out the programme by appropriately remunerated posts/stipendiary positions or other ways of financing for trainees. (B 4.5.1)
- ensure participation by the trainee in all medical activities - including on-call duties - relevant for the education. (B 4.5.2)
- define and make known the service conditions and responsibilities of trainees. (B 4.5.3)
- replace interruptions of training caused by pregnancy (including maternity/paternity leave), sickness, military service or secondment by additional training. (B 4.5.4)

Quality development standards:
The programme provider(s) **should**

- ensure that the service components of trainee positions are not dominating. (Q 4.5.1)
- take into account the needs of the patients, continuity of care and the educational needs of the trainee in the structuring of duty hours and on-call schedules. (Q 4.5.2)

- allow part-time education under special circumstances, structured according to an individually tailored programme and the service background. (Q 4.5.3)
- ensure that the total duration and quality of part-time education is not less than those of full-time trainees. (Q 4.5.4)

Annotations:

- *Remunerated posts/stipendiary positions* refer to contractual service positions and would include internship, residency and higher specialist training.
- *Other ways of financing* would include payment of tuition through private means, loans or institutional support.
- *Participation by the trainee in all medical activities* would mean devoting professional activities to practical training and theoretical learning.
- *Service conditions and responsibilities* would include appropriate supervision and limitation of risks to the safety of patient, trainees and trainers.
- *The service components of trainee positions* would include clinical workload without further learning value, and would be subject to definitions and protections embodied in a contract.

5. Trainers

5.1. RECRUITMENT AND SELECTION POLICY

Basic standards:
The programme provider(s) **must**

- formulate and implement a recruitment and selection policy for trainers, supervisors and teachers that specifies
  - the expertise required. (B 5.1.1)
  - criteria for scientific, educational and clinical merit, including the balance between teaching, research and service qualifications. (B 5.1.2)
  - their responsibilities. (B 5.1.3)
  - the duties of the training staff and specifically the balance between educational, research and service functions. (B 5.1.4)
- in its selection policy take into account the mission of the programme, the needs of the education system and the needs of the health care system. (B 5.1.5)

Quality development standards:
The programme provider(s) **should**

- in the formulation and implementation of its staff policy
  - recognise the responsibility of all physicians as part of their professional obligations to participate in the practice-based postgraduate education of medical doctors. (Q 5.1.1)
  - reward participation in postgraduate education. (Q 5.1.2)
  - ensure that trainers are current in the relevant field. (Q 5.1.3)
  - ensure that trainers with a sub-speciality function are approved for relevant specific periods during the education and for other periods of education dependent on their qualifications. (Q 5.1.4)
  - reward participation in programmes for developing their educational expertise. (Q 5.1.5)
  - engage educational expertise in trainer development. (Q 5.1.6)

Annotations:

- *Recruitment and selection policy* would include ensuring a sufficient number of highly qualified clinicians, health care managers and scientists to deliver the programme.
- *Trainers, supervisors and teachers* would comprise inter-professional trainers and not only physicians.
- *Expertise* would include recognition as a specialist in the relevant field of medicine. Expertise should be defined and checked regularly.
- *Training staff* would include physicians and other health personnel.
- *Service functions* would include clinical duties in the health care delivery system as well as participation in governance and management.

- *Current in the relevant field* implies that trainers have access to real pedagogical education and tutor/supervisor training.
- *Educational expertise,* cf. 6.6.

5.2. TRAINER OBLIGATIONS AND TRAINER DEVELOPMENT

Basic standards:
The programme provider(s) **must**

- ensure that trainers have time for teaching, supervision and learning. (B 5.2.1)
- provide faculty development of trainers and supervisors. (B 5.2.2)
- ensure periodic evaluation of trainers. (B 5.2.3)

Quality development standards:
The programme provider(s) **should**

- in the formulation and implementation of its staff policy
  - include in staff development support for trainers regarding teacher education and further professional development, both in their speciality and in educational expertise. (Q 5.2.1)
  - appraise and recognise meritorious academic activities in functions as trainers, supervisors and teachers. (Q 5.2.2)
  - define a ratio between the number of recognised trainers and the number of trainees ensuring close personal interaction and monitoring of the trainee. (Q 5.2.3)

Annotations:

- *Time for teaching, supervision and learning* would imply a balance between clinical work load and learning opportunities and would require coordination of work schedules.
- *Evaluation of trainers* would include feedback from the trainee to the trainer.
- *Recognition of meritorious academic activities* would be by rewards, promotion and/or remuneration.

6. Educational Resources

6.1. PHYSICAL FACILITIES

Basic standards:
The programme provider(s) **must**

- offer the trainee
  - space and opportunities for practical and theoretical study. (B 6.1.1)
  - access to up-to-date professional literature. (B 6.1.2)
  - adequate information and communication technology. (B 6.1.3)
  - equipment for training in practical techniques. (B 6.1.4)
  - a safe learning environment. (B 6.1.5)

Quality development standards:
The programme provider(s) **should**

- regularly update the physical facilities and equipment regarding their appropriateness and quality in relation to postgraduate education. (Q 6.1.1)

Annotations:

- *Physical facilities* of the training location would include lecture halls, class, group and tutorial rooms, teaching and research laboratories, clinical skills laboratories, offices, libraries, information technology facilities and trainee amenities such as adequate study space, on-call accommodation, personal storage lockers and recreational facilities, where these are appropriate.
- *A safe learning environment* would include provision of necessary information and protection from harmful substances, specimens and organisms, laboratory safety regulations and safety equipment.

6.2. LEARNING SETTINGS

Basic standards:
The programme provider(s) **must**

- select and approve the learning settings. (B 6.2.1)
- have access to
  - sufficient clinical/practical facilities to support the delivery of learning. (B 6.2.2)
  - a relevant number of patients. (B 6.2.3)
  - an appropriate case-mix of patients and patient materials to meet intended educational outcomes, including the use of both inpatient and outpatient (ambulatory) care and on-duty activity. (B 6.2.4)

Quality development standards:
The programme provider(s) **should**

- by the choice of learning settings ensure education
  - in promotion of health and prevention of disease. (Q 6.2.1)
  - in hospitals (general hospitals and, when relevant, academic teaching hospitals) and in community based facilities. (Q 6.2.2)

Annotations:

- *Learning settings* would include hospitals with adequate mix of primary, secondary and tertiary services and sufficient patient wards and diagnostic departments, laboratories, ambulatory services (including primary care), clinics, primary health care settings, health care centres, hospices and other community health care settings as well as skills laboratories, allowing clinical training to be organised using an appropriate mix of clinical settings and rotations throughout all relevant main disciplines.
- *Patients* would include validated simulation using standardised patients or other techniques, where appropriate, to complement, but not substitute clinical training.
- *Community-based facilities* would include primary health care centres or stations, speciality clinics, specialist practices, nursing homes and other facilities where health care is provided for a specific geographical area.

6.3. INFORMATION TECHNOLOGY

Basic standards:
The programme provider(s) **must**

- ensure access to web-based or other electronic media. (B 6.3.1)
- use information and communication technology in an effective and ethical way as an integrated part of the programme. (B 6.3.2)

Quality development standards:
The programme provider(s) **should**

- enable trainers and trainees to use existing and new information and communication technology for
  - self-directed learning. (Q 6.3.1)
  - communication with colleagues. (Q 6.3.2)
  - accessing relevant patient data and health care information systems. (Q 6.3.3)
  - patient/practice managements. (Q 6.3.4)

Annotations:

- *Effective use of information and communication technology* would include use of computers, cell/mobile telephones, internal and external networks and other means, as well as coordination with library services. The use of information and communication technology may be part of education for evidence-based medicine and in preparing the trainees for continuing medical education and professional development.
- *Ethical use* refers to the challenges to both physician and patient privacy and confidentiality following the advancement of technology in medical education and health care. Appropriate safeguards would be included in relevant policy to promote the safety of physicians and patients while empowering them to use new tools.

6.4. CLINICAL TEAMS

Basic standards:
The programme provider(s) **must**

- ensure experience of working in a team with colleagues and other health professionals. (B 6.4.1)

Quality development standards:
The programme provider(s) **should**

- encourage learning in a multi-disciplinary/multiprofessional team. (Q 6.4.1)
- promote development of ability to guide and teach other health professionals. (Q 6.4.2)

Annotations:

- *Working in a team* would foster multi-disciplinary learning.

6.5. MEDICAL RESEARCH AND SCHOLARSHIP

Basic standards:
The programme provider(s) **must**

- that the trainee achieves knowledge of and ability to apply the scientific basis and methods of the chosen field of medicine. (B 6.5.1)
- adequate integration and balance between training and research. (B 6.5.2)

Quality development standards:
The programme provider(s) **should**

- encourage trainees to engage in medical research and quality development of health and the health care system. (Q 6.5.1)
- provide sufficient time within the programme for trainees to undertake research. (Q 6.5.2)
- give access to research facilities and activities in the training settings. (Q 6.5.3)

Annotations:

- *Medical research and scholarship* encompasses scientific research in basic biomedical, clinical, behavioural and social sciences. Medical scholarship means the academic attainment of advanced medical knowledge and inquiry. The medical research basis of the programme would be ensured by research activities within the training settings or affiliated institutions and/or by the scholarship and scientific competencies of the trainer staff. Influences on current education would facilitate teaching of scientific methods and evidence-based medicine, cf. 2.2.
- Education in *scientific basis and methods* would include the use of elective research projects to be conducted by trainees.

6.6. EDUCATIONAL EXPERTISE

Basic standards:
The programme provider(s) **must**

- formulate and implement a policy on the use of educational expertise relevant in
  - programme planning. (B 6.6.1)
  - implementation of the programme. (B 6.6.2)
  - evaluation of the programme. (B 6.6.3)

Quality development standards:
The programme provider(s) **should**

- pay attention to the development of expertise in educational evaluation and in research in the discipline of medical education. (Q 6.6.1)
- allow staff to pursue educational research interests. (Q 6.6.2)

Annotations:

- *Educational expertise* would deal with problems, processes and practices of postgraduate medical education and assessment, and would include medical doctors with experience in medical education, educational psychologists and sociologists with experience in medical education. It can be provided by an education unit or be acquired from another national or international institution.
- *Research in the discipline of medical education* investigates theoretical, practical and social issues in medical education.

6.7. LEARNING IN ALTERNATIVE SETTINGS

Basic standards:
The programme provider(s) **must**

- formulate and implement a policy on accessibility of individual trainees to education opportunities at alternative training settings within or outside the country. (B 6.7.1)
- establish a system for the transfer of the results of education. (B 6.7.2)

Quality development standards:
The programme provider(s) **should**

- facilitate regional and international exchange of trainers and trainees by providing appropriate resources. (Q 6.7.1)
- establish relations with corresponding national or international bodies with the purpose of facilitating exchange and mutual recognition of education elements. (Q 6.7.2)

Annotations:

- *Formulate and implement a policy* would include consultation with principal as well as other stakeholders, cf. 1.4, annotations.
- *Transfer of results of education* can be facilitated through active programme coordination and use of credits.

7. Programme Evaluation

7.1. MECHANISMS FOR PROGRAMME MONITORING AND EVALUATION

Basic standards:
The programme provider(s) **must**

- routinely monitor the programme. (B 7.1.1)
- establish and apply a mechanism for programme evaluation. (B 7.1.2)
- in the evaluation address
  - the mission, the intended as well as acquired educational outcomes, the educational programme, assessment, if any, the programme provider and the educational resources. (B 7.1.3)
  - the relation between the recruitment policy and the needs of the education and health systems. (B 7.1.4)
  - programme process. (B 7.1.5)
  - methods of assessment. (B 7.1.6)
  - progress of trainees. (B 7.1.7)
  - trainer qualifications. (B 7.1.8)
  - identified concerns. (B 7.1.9)
- ensure that relevant results of evaluation influence the programme. (B 7.1.10)
- involve principal stakeholders in evaluation. (B 7.1.11)

Quality development standards:
The programme provider(s) **should**

- make the process and results of evaluation transparent to principal as well as other stakeholders. (Q 7.1.1)

Annotations:

- *Programme monitoring* would imply the routine collection of data about key aspects of the programme for the purpose of ensuring that the education is on track and for identifying any areas in need of intervention. The collection of data is often part of the administrative procedures in connection with admission of trainees, assessment and completion of the programme.

- *Programme evaluation* is the process of systematically gathering information to judge the effectiveness and adequacy of the education programme, using monitored data, collected feedback and results of special evaluation studies. This would imply the use of reliable and valid methods of data collection and analysis for the purpose of demonstrating the qualities of the education in relation to the mission and the intended and acquired educational outcomes. It would include information about average actual duration of education, scores, pass and failure rates at examinations, success- and dropout rates, as well as time spent by the trainees on areas of special interest.
  Involvement of external reviewers from outside the programme and the institution as well as experts in medical education and evaluation and regulatory bodies would further broaden the quality of postgraduate education.
- *Programme process* in this document is used synonymously with curriculum model. It covers framework and content/syllabus.
- *Identified concerns* would include insufficient fulfilment of intended educational outcomes. It would use measures of and information about intended educational outcomes, including identified weaknesses and problems, as feedback to conduction of interventions and plans for corrective action, programme development and improvements; this requires a safe and supporting environment for feedback by trainers and trainees.
- *Principal as well as other stakeholders,* cf. 1.4, annotation.

7.2. TRAINER AND TRAINEE FEEDBACK

Basic standards:
The programme provider(s) **must**

- Seek feedback about programmes from
  - trainers. (B 7.2.1)
  - trainees. (B 7.2.2)
  - employers. (B 7.2.3)

Quality development standards:
The programme provider(s) **should**

- actively involve trainers and trainees in planning programme evaluation and in using its results for programme development. (Q 7.2.1)

Annotations:

- *Feedback* would include trainees' reports and other information about the processes and products of the educational programmes. It would also include information about malpractice or inappropriate conduct by teachers or trainees with or without legal consequences.

7.3. PERFORMANCE OF QUALIFIED DOCTORS

Basic standards:
The programme provider(s) **must**

- routinely monitor performance of qualified doctors. (B 7.3.1)
- seek feedback on performance of qualified doctors from employers. (B 7.3.2)
- establish and apply a mechanism for programme evaluation using collected data on performance of qualified doctors. (B 7.3.3)

Quality development standards:
The programme provider(s) **should**

- inform about the results of the evaluation of the performance of qualified doctors to those responsible for
  - selection of trainees. (Q 7.3.1)
  - programme planning. (Q 7.3.2)

Annotations:

- *Performance of qualified doctors* would cover long-term acquired outcomes and would be measured e.g. by results of national specialist examinations, benchmarking procedures, international examinations or career development. It would, while avoiding the risk of programme uniformity, provide a basis for programme improvement.
- *Qualified doctors* means doctors having completed postgraduate medical education.
- *Collected data* would besides monitored data and connected feedback also include results of special studies of performance.

7.4. INVOLVEMENT OF STAKEHOLDERS

Basic standards:
The programme provider(s) **must**

- involve the principal stakeholders in its programme for monitoring and evaluation. (B 7.4.1)

Quality development standards:
The programme provider(s) **should**

- for other stakeholders
  - allow access to results of course and programme evaluation. (Q 7.4.1)
  - seek their feedback on the performance of doctors. (Q 7.4.2)
  - seek their feedback on the programme. (Q 7.4.3)

Annotations:

- *Principal stakeholders,* cf. 1.4, annotation.
- *Other stakeholders*, cf. 1.4, annotation.

8. Governance and Administration

8.1. GOVERNANCE

Basic standards:
The programme provider(s) **must**

- ensure that the programme is conducted in accordance with regulations concerning
  - admission of trainees (selection criteria and number). (B 8.1.1)
  - process. (B 8.1.2)
  - assessment. (B 8.1.3
  - intended educational outcomes. (B 8.1.4)
- document completion of education by the issue of degrees, diplomas, certificates or other evidence of formal qualifications for use by both national and international authorities. (B 8.1.5)
- be responsible for a programme for quality development. (B 8.1.6)

Quality development standards:
The programme provider(s) **should** ensure

- transparency of the work of governance and its decisions. (Q 8.1.1)
- adequacy of the programme to the health needs of the population it serves. (Q 8.1.2)

Annotations:

- *Governance* means the act and/or the structure of governing the programme and the involved institutions. Governance is primarily concerned with policy making, the processes of establishing institutional and programme policies and also with control of the implementation of the policies. The institutional and programme policies would normally encompass decisions on the mission of the programme, admission policy, staff recruitment and selection policy and decisions on interaction and linkage with medical practice and the health sector as well as other external relations.
- *Completion of education* would - depending on the level of education - result in a doctor with the right to independent practice, including medical specialists or medical experts.
- *Transparency* would be obtained by newsletters, web-information or disclosure of minutes.

8.2. ACADEMIC LEADERSHIP

Basic standards:
The programme provider(s) **must**

- take responsibility for the leadership/staff and organisation of postgraduate medical education. (B 8.2.1).

Quality development standards:
The programme provider(s) **should**

- evaluate the leadership/staff at defined intervals with respect to
  - the mission of the programme. (Q 8.2.1)
  - the acquired outcomes of the programme. (Q 8.2.2)

Annotations:

- *Leadership/staff* refers to the positions and persons within the governance and management structures being responsible for decisions on professional matters in programme implementation, teaching and assessment.
- *Evaluate the leadership/staff* would involve consultation of external reviewers.

8.3. EDUCATIONAL BUDGET AND RESOURCE ALLOCATION

Basic standards:
The programme provider(s) **must**

- define responsibility and authority for managing the budgets of the programme. (B 8.3.1)
- allocate the resources necessary for the implementation of the programme and distribute the educational resources in relation to educational needs. (B 8.3.2)

Quality development standards:
The programme provider(s) **should**

- manage the budget in a way that supports
  - the service obligations of trainers and trainees. (Q 8.3.1)
  - innovations in the programme. (Q 8.3.2)

Annotations:

- *The educational budget* would depend on the budgetary practice in the country and would be linked to a transparent budgetary plan for the programme.

8.4. ADMINISTRATION AND MANAGEMENT

Basic standards:
The programme provider(s) **must**

- have an administrative and professional staff that is appropriate to
  - support implementation of the educational programme and related activities. (B 8.4.1)
  - ensure good management and resource deployment. (B 8.4.2)

Quality development standards:
The programme provider(s) **should**

- include an internal programme of quality assurance of the management, including regular review. (Q 8.4.1)
- ensure that management submits itself to regular review to achieve quality improvement. (Q 8.4.2)

Annotations:

- *Administrative and professional staff* in this document refers to the positions and persons within the governance and management structures being responsible for the administrative support to policy making and implementation of policies and plans and would - depending on the organisational structure of the administration - include head and staff in the programme secretariat, heads of financial administration, staff of the budget and accounting offices, officers and staff in the admissions office and heads and staff of the departments for planning, personnel and IT.
- *Management* means the act and/or the structure concerned primarily with the implementation of institutional and programme policies including the economic and organisational implications, i.e. the actual allocation and use of resources in the programme. Implementation of institutional and programme policies would involve carrying into effect the policies and plans regarding mission, the programme, admission, staff recruitment and external relations.
- *Internal programme of quality assurance* would include consideration of the need for improvements and review of the management.
- *Regular review* would be conducted by institutional organisations external to and independent of the provider.

8.5. REQUIREMENTS AND REGULATIONS

Basic standards:
The programme provider(s) **must**

- follow the definition by a national authority of the number and types of recognised medical specialities and other medical expert functions for which approved education programmes are developed. (B 8.5.1)

Quality development standards:
The programme provider(s) **should**

- define programmes for approved postgraduate medical education in collaboration with stakeholders. (Q 8.5.1)

Annotations:

- *A national authority* with responsibility for postgraduate medical education would be established according to national laws and regulations and would be a governmental unit, an organisation or another regulatory or professional body.
- *Stakeholders* would include principal as well as other stakeholders, cf. 1.4, annotation.

9. Continuous Renewal

Basic standards:
In realising the dynamics of postgraduate medical education and involvement of the relevant stakeholders, and in order to ensure sustainable quality
the programme provider(s) **must**

- initiate procedures for regularly reviewing and updating the process, structure, content, outcomes/competencies, assessment and learning environment of the programme. (B 9.0.1)
- rectify documented deficiencies. (B 9.0.2)
- allocate resources for continuous renewal. (B 9.0.3)

Quality development standards:
The programme provider(s) **should**

- base the process of renewal on prospective studies and analyses and on results of local evaluation and the medical education literature. (Q 9.0.1)
- ensure that the process of renewal and restructuring leads to the revision of the policies and practices of postgraduate medical education programmes in accordance with past experience, present activities and future perspectives. (Q 9.0.2)
- address the following issues in its process of renewal:
  - adaptation of mission statement of postgraduate medical education to the scientific, socio-economic and cultural development of the society. (Q 9.0.3) (cf. 1.1)
  - modification of the intended outcomes required at completion of postgraduate education in the chosen field of medicine in accordance with documented needs of the community that the newly trained doctor will enter. (Q 9.0.4) (cf. 1.3)
  - adaptation of the learning approaches and education methods to ensure that these are appropriate and relevant. (Q 9.0.5) (cf. 2.1)
  - adjustment of the structure, content and duration of postgraduate medical education programmes in keeping with developments in the basic biomedical sciences, the behavioural and social sciences, the clinical sciences, changes in the demographic profile and health/disease pattern of the population, and socio-economic and cultural conditions. The adjustment would ensure that new relevant knowledge, concepts and methods are included and outdated ones discarded. (Q 9.0.6) (cf. 2.4)
  - development of assessment principles and methods according to changes in intended outcomes and instructional methods.(Q 9.0.7) (cf. 3.1 and 3.2)
  - adaptation of trainee recruitment policy, selection methods and trainee intake to changing expectations and circumstances, human resource needs, changes in the basic medical education and the requirements of the programme. (Q 9.0.8) (cf. 4.1 and 4.2)
  - adaptation of trainer, supervisor and teacher recruitment and development policy according to changing needs in postgraduate medical education. (Q 9.0.9) (cf. 5.1 and 5.2)
  - updating of training settings and other educational resources to changing needs in postgraduate medical education, i.e. the number of trainees, number and profile of trainers, the education programme and accepted contemporary education principles. (Q 9.0.10) (cf. 6.1-6.3)
  - refinement of the process of programme monitoring and evaluation. (Q 9.0.11) (cf. 7.1-7.4)

- development of the organisational structure and of governance and management to cope with changing circumstances and needs in postgraduate medical education and, over time, accommodating the interests of the different groups of stakeholders. (Q 9.0.12) (cf. 8.1-8.5)

Annotations:

- *Prospective studies* would include research and studies to collect and generate data and evidence on country-specific experiences with best practice.

Bibliography

The bibliography covers publications and documents that provide background and development of the WFME standards and links to present pages, illustrating, without being complete, present (as of September 2015) development of the medical education standard field.

PREVIOUS AND PRESENT WFME STANDARDS

World Federation of Medical Education (WFME). Basic Medical Education. WFME Global Standards for Quality Improvement [Internet]. 2003.
Available from:
http://wfme.org/standards/bme/3-quality-improvement-in-basic-medical-education-english/file

World Federation of Medical Education (WFME). Basic Medical Education. WFME Global Standards for Quality Improvement. New edition [Internet]. 2012.
Available from:
http://wfme.org/standards/bme/78-new-version-2012-quality-improvement-in-basic-medical-education-english/file

World Federation of Medical Education (WFME). Basic Medical Education. WFME Global Standards for Quality Improvement. New edition 2012, revised. 2015

World Federation for Medical Education (WFME). Postgraduate Medical Education. WFME Global Standards for Quality Improvement [Internet]. 2003.
Available from:
http://wfme.org/standards/pgme/17-quality-improvement-in-postgraduate-medical-education-english/file

World Federation for Medical Education (WFME). Postgraduate Medical Education. WFME Global Standards for Quality Improvement. New edition. 2015.

World Federation for Medical Education (WFME). Continuing Professional Development of Medical Doctors. WFME Global Standards for Quality Improvement [Internet]. 2003.
Available from:
http://wfme.org/standards/cpd/16-continuing-professional-development-cpd-of-medical-doctors-english/file

World Federation for Medical Education (WFME). Continuing Professional Development of Medical Doctors. WFME Global Standards for Quality Improvement. New edition. 2015.

World Federation for Medical Education (WFME) and The Association of Medical Schools in Europe (AMSE). WFME Global Standards for Quality Improvement in Medical Education. European Specifications [Internet]. 2007.
Available from:
http://wfme.org/standards/european-specifications/21-european-specifications-english/file

GENERAL

Boelen C, Bandaranayake R, Bouhuijs PAJ, Page GG, Rothman AI. Towards the Assessment of Quality in Medical Education [Internet]. WHO/HRH/92.7;1992.
Available from:
http://whqlibdoc.who.int/hq/1992/WHO_HRH_92.7.pdf

Christensen L, Karle H, Nystrup J. Process-outcome interrerelationship and standard setting in medical education: the need for a comprehensive approach. Med Teach. 2007;29:672-7.

Cohen J. Defining international standards in basic medical education: the World Federation for Medical Education has initiated a timely discussion. Commentary. Med Educ. 2000;34:600-1.

Cohen J. Academic medicine's latest imperative: achieving better health care through global medical education standards. Med Educ. 2003;37:950-1.

European Commission. Directive 2013/55/EU of the European Parliament and of the Council [Internet]. 2013.
Available from:
http://eur-lex.europa.eu/legal-content/EN/TXT/?uri=CELEX:32013L0055

Gastel BA. Toward a global consensus on the quality of medical education: serving the needs of populations and individuals. Acad Med. 1995;70 (Suppl 7):S73-5.

General Medical Council (GMC). Good medical practice [Internet]. 2013 with update 2014. Available from:
www.gmc-uk.org/guidance/good_medical_practice.asp

Gordon D, Christensen L, Dayrit M, Dela F, Karle H, Mercer H. Educating health professionals: the Avicenna project. Lancet. 2008;371:966-7.

Gordon D, Christensen L, Karle H, Walters T. The Avicenna Directories – A new tool in quality assurance of medical education. World Medical Journal. 2009;55:9-11.

Hays R. International accreditation of medical schools. Letter to the Editor. Med Educ. 2003;37:662.

Hays R. and Baravilala M. Applying global standards across national boundaries: lessons learned from an Asia-Pacific example. Commentary. Med Educ. 2004;38:582-4

Karle H. Global standards in medical education for better health care. Commentary. Med Educ. 2002;36:1116.

Karle H. Global Standards and Accreditation in Medical Education : A view from the WFME. Acad Med. 2006;81(Suppl 12):S43-8.

Karle H and on the behalf of the Executive Council World Federation for Medical Education. International recognition of basic medical education programmes: A WFME Position Paper. Med Educ. 2008;42:12-7.

Karle H, Walton H, Lindgren S. The World Federation for Medical Education (WFME). History of the First Forty Years 1972-2012 [Internet]. 2012.
Available from:
http://wfme.org/documents/about-wfme/79-wfme-history-of-the-first-forty-years-1972-2012/file

Lilley PM, Harden RM. Standards and medical education. Editorial. Med Teach. 2003;25:349-51.

Lindgren S, Karle H. Social accountability of medical education: Aspects on global accreditation. Med Teach. 2011;33:667-72.

van Niekerk JP. WFME Global Standards receive ringing endorsement. Commentary. Med Educ. 2003;37:585-6.

van Niekerk JP, Christensen L, Karle H, Lindgren S and Nystrup J. WFME Global Standards in Medical Education: status and perspectives following the 2003 WFME World Conference. Conference Report. Med Educ. 2003;37:1050-4.

Organisation for PhD Education in Biomedicine and Health Sciences in the European System (ORPHEUS), Association of Medical Schools in Europe (AMSE) and World Federation of Medical Education (WFME). Standards for PhD Education in Biomedicine and Health Sciences in Europe. A proposal [Internet]. 2012.
Available from:
http://wfme.org/standards/phd/57-standards-for-phd-education-in-biomedicine-and-health-sciences-in-europe/file

Prideaux D. Globalisation and Health Professional Education. Editorial. The ANZAME Journal "Focus on Health Professional Education: A Multi-Disciplinary Journal". 2003;5:V.

Royal College of Physicians and Surgeons of Canada. CanMEDS 2005 Framework [Internet]. 2005.
Available from:
http://www.royalcollege.ca/portal/page/portal/rc/common/documents/canmeds/framework/the_7_canmeds_roles_e.pdf

Royal College of Physicians and Surgeons of Canada. The Draft CanMEDS 2015 Physicians Competency Framework [Internet]. Frank JR, Snell LS, Sherbino J, editors. 2015.
Available from:
http://www.royalcollege.ca/portal/page/portal/rc/common/documents/canmeds/framework/canmeds2015_framework_series_III_e.pdf.

Segouin C, Nystrup J, Christensen L, Karle H. Faut-il prescrire des standards internationaux en éducation médicale? Compte-rendu de la conference mondiale organisée par la World Federation for Medical Education (WFME) à Copenhague (15-19 Mars 2003) "Global standards in medical education for better health care". Pédagogie Médicale. 2004;5:1-3.

The Edinburgh Declaration. Notes and Views. The Lancet. 1988;8068:464.

World Federation for Medical Education (WFME). Proceedings of the World Summit on Medical Education. Med Educ. 1994;28 (Suppl s1):1-171.

World Federation for Medical Education (WFME). The Executive Council. International standards in medical education: assessment and accreditation of medical schools' educational programmes. Med Educ.1998;32:549-58.

WFME World Conference "Global Standards for Medical Education – For BetterHealth Care". Copenhagen 15-19 March 2003.

World Federtion for Medical Education (WFME) and Federation for Advancement of International Medical Eduction and Research (FAIMER). New World Directory of Medical Schools. In collaboration with WHO and the University of Copenhagen [Internet]. 2014. Available from:
http://www.wdoms.org

World Health Organization (WHO).World Health Assembly (WHA). WHA Resolution 42.38; 1989.

World Health Organization (WHO). World Health Assembly (WHA). Reorientation of medical education and medical practice for health for all [Internet]. WHA Resolution 48.8;1995. Available from:
http://www.who.int/hrh/resources/WHA48-8_EN.pdf?ua=1

World Health Organization (WHO). Division of Development of Human Ressources. Changing Medical Education. An Agenda for Action [Internet]. WHOEDUC/91.200;1991. Available from:
http://apps.who.int/iris/bitstream/10665/60494/1/WHO_EDUC_91.200.pdf?ua=1

World Health Organization (WHO). Division of Development of Human Ressources. Priorities at the interface of health care, medical practice and medical education: Abbreviated report of the global conference on international collaboration on medical education and practice [Internet]. WHO/HRH/95.3;1994.
Available from:
http://apps.who.int/iris/bitstream/10665/59554/1/WHO_HRH_95.3.pdf

World Health Organization (WHO). Doctors for health. A WHO global strategy for changing medical education and medical practice for health for all [Internet]. WHO/HRH/96.1;1996. Available from:
http://whqlibdoc.who.int/hq/1996/WHO_HRH_96.1.pdf

World Health Organization (WHO). World Directory of Medical Schools, 7th ed. Geneva; 2000.

World Health Organization (WHO). Department of Human Resources for Health. Global standards for the initial education of professional nurses and midwives [Internet]. 2009. Available from:
http://www.who.int/hrh/nursing_midwifery/hrh_global_standards_education.pdf

World Health Organization (WHO) and World Federation for Medical Education (WFME). WHO/WFME strategic partnership to improve medical education [Internet]. 2004. Available from:
http://who.int/hrh/links/partnership/en/

World Health Organization (WHO), Regional Office for Eastern Mediterranean (WHO/EMRO) and World Federation for Medical Education, Association for Medical Education in the Eastern Mediterranean (WFME-AMEEMR). Regional consultation on accreditation of health professions education [Internet]. WHO-EM/HRH/629/E;2011.
Available from:
http://apps.who.int/iris/bitstream/10665/116094/1/IC_Meet_Rep_2011_EN_14538.pdf?ua=1

World Medical Association (WMA). Medical Ethics Manual, 2nd ed [Internet]. 2009.
Available from:
http://www.wma.net/en/30publications/30ethicsmanual/pdf/ethics_manual_en.pdf

PME-RELATED

American Board of Medical Specialties (ABMS). A Trusted Credential Based on Core Competencies [Internet]. 1999.
Available from:
http://www.abms.org/board-certification/a-trusted-credential/based-on-core-competencies/

Australian Medical Council Limited (AMC). Assessing specialist medical education and training [Internet]. 2015.
Available from:
http://www.amc.org.au/accreditation/medical-education

European Commsission. Fourth Report and Recommendations on the Conditions for Specialist Training. Doc. XV/E/8306/3/96-EN;1997.

European Union of Medical Specialists (UEMS). European Standards in Medical Training. Charter on Training of Medical Specialists in the European Community [Internet]. 1994.
Available from:
http://www.uems.eu/areas-of-expertise/postgraduate-training/european-standards-in-medical-training

Royal College of Physicians and Surgeons of Canada. Royal College Discipline Recognition [Internet].
Available from:
http://www.royalcollege.ca/portal/page/portal/rc/credentials/discipline_recognition

Standing Committee of European Doctors. Quality improvement in postgraduate medical education [Internet].
Available from: http://cpme.eu/policies/page12

Appendix: Members of Task Force

WORKING PARTY

Professor Leif Christensen
Senior Advisor
World Federation for Medical Education
University of Copenhagen
Copenhagen, Denmark

Professor Janet Grant
Education in Medicine, the Open University
Director, Centre for Medical Education in Context
Special Advisor
World Federation for Medical Education
London, UK

Dr. Hans Karle (Chairman)
President emeritus
World Federation for Medical Education
University of Copenhagen
Copenhagen, Denmark

Professor Stefan Lindgren
Past President
World Federation for Medical Education
Lund University
Malmö, Sweden

Dr. Jørgen Nystrup
Senior Advisor
World Federation for Medical Education
University of Copenhagen
Copenhagen, Denmark

Professor Hans Sjöström
Senior Advisor
World Federation for Medical Education
University of Copenhagen
Copenhagen, Denmark

INTERNATIONAL PANEL OF EXPERTS

(The list comprises contributors to the 2003 document as well as to the revised document)

Dr. Walid A. Abubaker
Regional Advisor
Educational Development & Training
Health Systems and Services Development
World Health Organization
Regional Office for the Eastern Mediterranean
Cairo, Egypt

Mrs. Brownell Anderson
Senior director for educational affairs
Association of American Medical Colleges
Washington, DC, USA

Dr. Carol A. Aschenbrener
Executive Vice President
Association of American Medical Colleges
Washington, DC, USA

Dr. Nadia Badrawi
Board Member of the National Authority for Quality Assurance and Accreditation in Education
President of the Arab Network for Quality Assurance in Higher Education
Cairo, Egypt

Dr. Sekelani S. Banda
School of Medicine
The University of Zambia
Lusaka, Zambia

Professor Raja Bandaranayake
20 Strickland Street
Rose Bay, NSW
Sydney, Australia

Professor Cheng Boji
Peking University Health Science Centre
Beijing, P.R. China

Professor Maria Rosa Fenoll Brunet
Universitat Rovira i Virgili
Facultat de Medicina i Ciències de la Salut
Dept. Ciències Mèdiques Bàsiques
Unitat d'Histologia i Neurobiologia
Reus, Spain

Dr. Francisco Campos
National Secretary of Labor and Education
Management in Health, Ministry of Health
Sao Paulo, Brazil

Professor Thomas V. Chacko
Director, PSG-FAIMER South Asia Regional Institute,
President, Tamil Nadu Voluntary Health Association,
Prof & Head, Community Medicine & Medical Education,
PSG Institute of Medical Sciences & Research
Coimbatore, India

Professor Jean-François Denef
Université Catholique de Louvain
Faculté de Médecine et Médecine Dentaire
Louvain-la-Neuve, Belgique

Professor Peter Dieter
Dean of Medical Education
Basic Science Years Institute of Physiological Chemistry
Faculty of Medicine
Dresden, Germany

Professor David Ellwood
Professor of Gynaecology and Obstetrics
Deputy Dean of the ANU Medical School
Chair of the AMC's Medical School Accreditation Committee
Canberra, Australia

Professor Michael Field
Medicine, Northern Clinical School
The University of Sydney
Sydney, Australia

Professor Laurie Geffen
East Brisbane, Australia

Ioana Goganau
Medical Student
Director for Medical Education
International Federation of Medical Students' Associations
Romania

Professor Richard Hays
Faculty of Health Sciences and Medicine
Bond University
Robina Queensland, Australia

Professor Susan R. Johnson
Obstetrics & Gynecology and Epidemiology
The University of Iowa
Iowa City, USA

Dr. Anne M. Keane
Head of Education and Training
Medical Council
Dublin, Ireland

Dr. Juan Victor Lara
Decano de la Coordinación de Medicina
Universidad de Guadalajara
Guadalajra, Jalisco, México

Professor Petr F. Livitckiy
Vice-Rector
I.M. Sechenov Moscow Medical Academy
Moscow, Russia

Professor Oleg Medvedev
Chair of Pharmacology
School of Medicine
Lomonosov Moscow State University
Moscow, Russia

Hugo Mercer
Deputy Editor
Latin American School of Social Sciences
FLACSO
Buenos Aires, Argentina

Professor Jadwiga Mirecka
Department of Medical Education
Jagiellonian University Medical College
Krakow, Poland

Professor Mariela Montilva
Dean of Medicine
Universidad Centroccidental Lisandro Alvarado
Estado Lara, Venezuela

Professor Richard Murray
Dean and Head of School
School of Medicine and Dentistry
James Cook University
Townsville, Australia

Dr. Tewfik Nawar
Conférence Internationale des Doyens
et Facultés de Médecine d'Expression Française
Faculté de Médecine
Université de Sherbrooke
Sherbrooke, Québec, Canada

Professor J.P. van Niekerk
HMPG Managing Editor
Rondebosch, South Africa

Dr. John J. Norcini
President and CEO
Foundation for Advancement of International
Medical Education and Research
Philadelphia, USA

Dr. Farida Nurmanbetova
National Accreditation Center
19, Imanova Street
Astana, Kazakhstan

Dr. Katrin Meyer
Wissenschaftliche Mitarbeiterin
Organ für Akkreditierung und Qualitätssicherung
der Schweizerischen Hochschulen
Falkenplatz 9, Postfach
Bern, Switzerland

Professor Gordon Page
Academic Director of the CCP
Department of Medicine
The University of British Columbia
Vancouver, BC, Canada

Mrs. Lorna M. Parkins
Executive Director
Caribbean Accreditation Authority for
Education in Medicine and Other Health
Professions
Kingston, Jamaica

Dr. Elaheh Malakan Rad
Associate professor of pediatric cardiology
Children's Medical Center
Affiliated to Tehran University of Medical Sciences
Tehran, Iran

Professor Herman van Rossum
Free University of Amsterdam
Amsterdam, the Netherlands

Professor Iskender Sayek
Department of General Surgery
Faculty of Medicine
Hacettepe University
Ankara, Turkey

Dr. Fernando Sanchez Jnr
Vice President for Academic Affairs
University of the East
Quezon City, Philippines

Dr. Christophe Segouin
Faculty of Medicine
University Paris 7 Denis Diderot
Paris, France

Dr. Nantana Sirisup
Faculty of Medicine
Chulalongkorn University
Bangkok, Thailand

Mrs. Theanne Walters
Deputy Chief Executive Officer.
Australian Medical Council
Kingston, Australia

Margot Weggemans
Medical Student
Liaison Officer for Medical Education Issues
International Federation of Medical Students' Associations
Ferney-Voltaire, France

Professor Robert F. Woollard
Dept of Family Practice
Faculty of Medicine University of British Columbia
Vancouver, BC, Canada

WFME Executive Council

Chairman:

Professor David Gordon
President
World Federation for Medical Education (WFME)
Ferney-Voltaire, France

Members:

Professor Ibrahim Al Alwan
President
Association for Medical Education
in the Eastern Mediterranean Region (AMEEMR)
Riyadh, Saudi Arabia

Dr. Emmanuel G. Cassimatis
President & CEO
Educational Commission for Foreign Medical Graduates (ECFMG)
Philadelphia, USA

Stijntje Dijk
Medical student
Liaison Officer for Medical Education Issues
International Federation of Medical Students' Associations (IFMSA)
Ferney-Voltaire, France

Professor Michael Field
President
Association for Medical Education
in the Western Pacific Region (AMEWPR)
Sidney, Australia

Dr. Otmar Kloiber
Secretary General
World Medical Association (WMA)
Ferney-Voltaire, France

Professor Stefan Lindgren
Past President
World Federation for Medical Education
Malmoe, Sweden

Dr. Pablo Pulido
President
Panamerican Federation of Associations of Medical Schools (PAFAMS)
Caracas, Venezuela

Professor Trudie Roberts
President
Association for Medical Education in Europe (AMEE)
Leeds, United Kingdom

Professor Nelson Sewankambo
President
Association of Medical Schools in Africa (AMSA)
Kampala, Uganda

Professor Rita Sood
President
South East Asian Regional Association for Medical Education (SEARAME)
New Delhi, India

Dr. Erica Wheeler
Technical Officer
Human Resources for Health
Department of Health Systems Policies and Workforce (HPW)
World Health Organization (WHO)
Geneva, Switzerland

WFME 18.IX.2015
