# Supplementary material for: The WFME global standards for quality improvement of postgraduate medical education: Which standards are also applicable in Germany? Recommendations for physicians with a license for postgraduate training and training agents
Source: GMS J Med Educ. 2022 Sep 15;39(4):Doc42. doi: 10.3205/zma001563 (PMC9585417; doi:10.3205/zma001563)
Supplement: WFME standards for quality improvement of postgraduate medical education recommended for physicians with a license for postgraduate training (PLT) [file JME-39-42-s-002.pdf]

Attachment 2: WFME[1] Standards for Quality Improvement of Postgraduate Medical Education recommended for physicians with a license for postgraduate training (PLT) – Part 1		
**No.**	**Standard**	**#[2]**
**from category 1. Mission and Outcomes**		
**The PLT <u>must</u> present the entire training program with its theoretical and practice-based components – with emphasis on the latter – resulting in a medical doctor who**		
1	- is competent to undertake comprehensive appropriate medical practice in the defined field of medicine.	B 1.1.6
2	- is capable of working in a professional manner.	B 1.1.7
3	- is able to work unsupervised and independently.	B 1.1.8
4	- is able to work within a professional/interprofessional team when relevant.	B 1.1.9
5	- is committed and prepared to life-long learning and participation in continuing medical education/continuing professional development.	B 1.1.10
6	- ensures improvement of patient care that is appropriate, effective, compassionate and safe in dealing with health problems and promotion of health, including a patient-centered and holistic approach.	B 1.1.11
**The PLT <u>must</u>**		
7	- ensure that trainees have appropriate working conditions to maintain their own health.	B 1.1.12
8	- include professionalism in the education of doctors.	B 1.2.1
9	- foster the professional autonomy necessary to enable the doctor to act in the best interests of the patient and the community.	B 1.2. 2
**from category 2. Framework of the Postgraduate Medical Education Program**		
**The PLT <u>must</u>**		
10	- use practice-based training involving the personal participation of the trainee in the services and responsibilities of patient care.	B 2.1.4
11	- use instructional and learning methods that are appropriate and ensure integration of practical and theoretical components.	B 2.1.5
12	- deliver the program in accordance with principles of equality.	B 2.1.6
13	- guide the trainee by means of supervision and regular appraisal and feedback.	B 2.1.8

Note [1] WFME=World Federation for Medical Education, [2] Numbering of the subcategories in the original WFME document

Attachment 2: WFME[1] Standards for Quality Improvement of Postgraduate Medical Education recommended for physicians with a license for postgraduate training (PLT) – Part 2		
**No.**	**Standard**	**#[2]**
	**from category 2. Framework of the Postgraduate Medical Education Program (continued)**	
**The PLT <u>must</u>**		
14	- ensure that the trainee becomes able to use scientific reasoning.	B 2.2.2
15	- becomes familiar with evidence-based medicine through exposure to a broad range of relevant clinical/practical experience in different settings in the chosen field of medicine.	B 2.2.3
16	- organize the program with appropriate attention to patient safety and autonomy.	B 2.3.11
17	- integrate practice and theory.	B 2.4.3
18	- define responsibility and authority for organizing, coordinating, managing and evaluating the individual educational setting and process.	B 2.5.1
19	- plan the education to expose the trainee to a broad range of experiences in the chosen field of medicine.	B 2.5.3
20	- describe and respect the apprenticeship nature of professional development.	B 2.6.1
21	integrate training and service.	B 2.6.2
22	- ensure that training is complementary to and integrated with service demands.	B 2.6.3
**The PLT <u>must</u> include in the program clinical work and relevant theory or experience of**		
23	- basic biomedical, clinical, behavioral and social sciences and preventive medicine.	B 2.3.1
24	- clinical decision-making.	B 2.3.2
25	- communication skills.	B 2.3.3
26	- medical ethics.	B 2.3.4
27	- public health.	B 2.3.5
28	- medical jurisprudence and forensic medicine.	B 2.3.6
29	- managerial disciplines.	B 2.3.7
30	- patient safety.	B 2.3.8
31	- doctors' self-care.	B 2.3.9
32	- the interface with complementary medicine.	B 2.3.10
**The PLT <u>should</u>**		
33	- increase the degree of independent responsibility of the trainee as skills, knowledge and experience grow.	Q 2.1.1
34	- adjust the content to scientific developments.	Q 2.2.2

Note [1] WFME=World Federation for Medical Education, [2] Numbering of the subcategories in the original WFME document

Attachment 2: WFME[1] Standards for Quality Improvement of Postgraduate Medical Education recommended for physicians with a license for postgraduate training (PLT) – Part 3		
**No.**	**Standard**	**#[2]**
	**from category 3. Assessment of Trainees**	
The program provider <u>must</u> use assessment principles, methods and practices that		
35	- are clearly compatible with intended educational outcomes and instructional methods.	B 3.2.1
36	- ensure that the intended educational outcomes are met by the trainees.	B 3.2.2
37	- promote trainee learning.	B 3.2.3
38	- ensure adequacy and relevance of education.	B 3.2.4
39	- ensure timely, specific, constructive and fair feedback to trainees on the basis of assessment results.	B 3.2.5
**The program provider(s) should use assessment principles, methods and practices that**		
40	- encourage integrated learning.	Q 3.2.1
41	- encourage involvement of practical clinical work.	Q 3.2.2
42	- facilitate interprofessional education.	Q 3.2.3
	**from category 4. Trainees**	
**The PLT <u>must</u>**		
43	- ensure access to a system for academic counseling of trainees.	B 4.3.1
44	- base the academic counseling of trainees on monitoring the progress in education including reported unintended incidents.	B 4.3.2
45	- ensure confidentiality in relation to counseling and support.	B 4.3.5
46	- offer career guidance and planning.	B 4.3.6
47	- formulate and implement a policy on trainee representation and appropriate participation in the design of the program.	B 4.4.2
48	- formulate and implement a policy on trainee representation and appropriate participation in the planning of trainees' working conditions.	B 4.4.3
49	- carry out the program by appropriately remunerated posts/stipendiary positions or other ways of financing for trainees.	B 4.5.1
50	- ensure participation by the trainees in all medical activities – including on-call duties – relevant for the education.	B 4.5.2
51	- define and make known the service conditions and responsibilities of trainees.	B 4.5.3

Note [1] WFME=World Federation for Medical Education, [2] Numbering of the subcategories in the original WFME document

Attachment 2 to Schwill S, Kadmon M, Hahn EG, Kunisch R, Berberat PO, Fehr F, Hennel E. *The WFME global standards for quality improvement of postgraduate medical education: Which standards are also applicable in Germany? Recommendations for physicians with a license for postgraduate training and training agents*. GMS J Med Educ. 2022;39(4):Doc42. DOI: 10.3205/zma001563

Attachment 2: WFME[1] Standards for Quality Improvement of Postgraduate Medical Education recommended for physicians with a license for postgraduate training (PLT) – Part 4		
**No.**	**Standard**	**#[2]**
	**from category 4. Trainees (continued)**	
	**The PLT <u>should</u>**	
52	- provide support in case of a professional crisis.	Q 4.3.1
53	- ensure that the service components of trainee positions are not dominating.	Q 4.5.1
54	- take into account the needs of the patients, continuity of care and the educational needs of the trainee in the structuring of duty hours and on-call schedules.	Q 4.5.2
55	- allow part-time education under special circumstances, structured according to an individually tailored program and the service background.	Q 4.5.3
	**from category 5. Physicians with a license for postgraduate training (PLT) and other postgraduate training personnel: Training Agent (TA)[3]**	
	**The PLT <u>must</u>**	
56	- ensure that training agents have time for teaching, supervision and learning.	B 5.2.1
57	- provide faculty development of training agents and supervisors.	B 5.2.2
58	- ensure periodic evaluation of training agents.	B 5.2.3
	**When formulation and implementation their staff policy and development, the PLT <u>should</u>**	
59	- reward participation in postgraduate medical education.	Q 5.1.2
60	- ensure that training agents are current in the relevant field.	Q 5.1.3
61	- reward participation in programs for developing their educational expertise.	Q 5.1.5
62	- engage educational expertise in training agent development.	Q 5.1.6
63	- include staff development support for training agents regarding teacher education and further<br>professional development, both in their speciality and in educational expertise	Q 5.2.1
64	- appraise and recognize meritorious academic activities in functions as training agents, supervisors and teachers	Q 5.2.2
	**[3] Training agent (TA) = medical and non-medical personnel who are involved in postgraduate medical education through PLT**	

Note [1] WFME=World Federation for Medical Education, [2] Numbering of the subcategories in the original WFME document

Attachment 2 to Schwill S, Kadmon M, Hahn EG, Kunisch R, Berberat PO, Fehr F, Hennel E. *The WFME global standards for quality improvement of postgraduate medical education: Which standards are also applicable in Germany? Recommendations for physicians with a license for postgraduate training and training agents*. GMS J Med Educ. 2022;39(4):Doc42. DOI: 10.3205/zma001563

Attachment 2: WFME[1] Standards for Quality Improvement of Postgraduate Medical Education recommended for physicians with a license for postgraduate training (PLT) – Part 5		
**No.**	**Standard**	**#[2]**
	**from category 6. Educational Resources**	
	**The PLT must**	
65	- select and approve the learning settings.	B 6.2.1
66	- have access to sufficient clinical/practical facilities to support the delivery of learning.	B 6.2.2
67	- have access to a relevant number of patients.	B 6.2.3
68	- have access to an appropriate case-mix of patients and patient materials to meet intended educational outcomes, including the use of both inpatient and outpatient (ambulatory) care and on-duty activity.	B 6.2.4
69	- ensure access to web-based or other electronic media.	B 6.3.1
70	- use information and communication technology in an effective and ethical way as an integrated part of the program.	B 6.3.2
71	- ensure experience of working in a team with colleagues and other health professionals.	B 6.4.1
72	- ensure that the trainee achieves knowledge of and ability to apply the scientific basis and methods of the chosen field of medicine.	B 6.5.1
	**The PLT should**	
73	- regularly update the physical facilities and equipment regarding their appropriateness and quality in relation to postgraduate education.	Q 6.1.1
74	- by the choice of learning settings ensure education in promotion of health and prevention of disease.	Q 6.2.1
75	- encourage learning in a multi-disciplinary/multi-professional team.	Q 6.4.1
76	- encourage trainees to engage in medical research and quality development of health and the health care system.	Q 6.5.1
	**The PLT should enable the training agent and the trainee to use current and new information and communication technology for**	
77	- self-directed learning.	Q 6.3.1
78	- communication with colleagues.	Q 6.3.2
79	- accessing relevant patient data and health care information systems.	Q 6.3.3

Note [1] WFME=World Federation for Medical Education, [2] Numbering of the subcategories in the original WFME document, TA = Training agent

Attachment 2 to Schwill S, Kadmon M, Hahn EG, Kunisch R, Berberat PO, Fehr F, Hennel E. *The WFME global standards for quality improvement of postgraduate medical education: Which standards are also applicable in Germany? Recommendations for physicians with a license for postgraduate training and training agents*. GMS J Med Educ. 2022;39(4):Doc42. DOI: 10.3205/zma001563

Attachment 2: WFME[1] Standards for Quality Improvement of Postgraduate Medical Education recommended for physicians with a license for postgraduate training (PLT) – Part 6		
**No.**	**Standard**	**#[2]**
	**from category 7. Postgraduate Training Evaluation**	
	**The PLT <u>must</u> seek feedback about the postgraduate training from**	
80	- training agents.	B 7.2.1
81	- trainees.	B 7.2.2
	**The PLT <u>should</u>**	
82	- actively involve training agents and trainees in planning program evaluation and in using its results for program development.	Q 7.2.1
	**from category 8. Governance and Administration**	
	**The PLT <u>must</u>**	
83	- define responsibility and authority for managing the budgets of the program.	B 8.3.1
84	- allocate the resources necessary for the implementation of the program and distribute the educational resources in relation to educational needs.	B 8.3.2
	**from category 9. Continuous Renewal**	
	**The PLT <u>must</u>**	
85	- initiate procedures for regularly reviewing and updating the process, structure, content, outcomes/competencies, assessment and learning environment of the program.	B 9.0.1
86	- rectify documented deficiencies.	B 9.0.2
87	- allocate resources for continuous renewal.	B 9.0.3
	**The PLT should deal with the following aspects of a continuous renewal process as part of the further development of the postgraduate training program:**	
88	- adaptation of mission statement of postgraduate medical education to the scientific, socio-economic and cultural development of the society. (cf 1.1)	
89	- adaptation of the learning approaches and education methods to ensure that these are appropriate and relevant. (cf 2.1)	Q 9.0.5
90	- updating of training settings and other educational resources to changing needs in postgraduate medical education, i.e. the number of trainees, number and profile of training agents, the education program and accepted contemporary education principles. (cf 6.1-6.3)	Q 9.0.10

Note [1] WFME=World Federation for Medical Education, [2] Numbering of the subcategories in the original WFME document, TA=Training agent

Attachment 2 to Schwill S, Kadmon M, Hahn EG, Kunisch R, Berberat PO, Fehr F, Hennel E. *The WFME global standards for quality improvement of postgraduate medical education: Which standards are also applicable in Germany? Recommendations for physicians with a license for postgraduate training and training agents*. GMS J Med Educ. 2022;39(4):Doc42. DOI: 10.3205/zma001563
